# Supplementary material for: PRPS2 enhances RNA m6A methylation by stimulating SAM synthesis through enzyme-dependent and independent mechanisms
Source: Nat Commun. 2025 Apr 28;16:3966. doi: 10.1038/s41467-025-59119-0 (PMC12037730; doi:10.1038/s41467-025-59119-0)
Supplement: Supplementary file 1 — Supplementary Information [file 41467_2025_59119_MOESM1_ESM.pdf]

SUPPLEMENTARY INFORMATION FOR

PRPS2 Enhances RNA m^{6}A Methylation by Stimulating SAM Synthesis Through Enzyme-Dependent and Independent Mechanisms

Lin Zhang[1,#], Xian Zhao[2,#], Jingyan Hu[1,#], Tingting Li[1], Hong-Zhuan Chen[3], Ao Zhang[4], Hao Wang[5,*], Jianxiu Yu[2,*], Liang Zhang[1,6,*]

[1]Department of Pharmacology and Chemical Biology, State Key Laboratory of Systems Medicine for Cancer, School of Medicine, Shanghai Jiao Tong University, Shanghai, 200025, China; [2]Department of Biochemistry and Molecular Cell Biology, Shanghai Key Laboratory of Tumor Microenvironment and Inflammation, Shanghai Jiao Tong University School of Medicine, Shanghai, 200025, China; [3]Institute of Interdisciplinary Integrative Biomedical Research, Shuguang Hospital, Shanghai University of Traditional Chinese Medicine, Shanghai, 201203, China; [4]Pharm-X Center, School of Pharmacy, Shanghai Jiao Tong University, Shanghai, 200240, China; [5]The Division of Thoracic Surgery, Zhongshan Hospital, Fudan University, Shanghai, 200032, China; [6]Department of Chemical Biology, School of Chemistry and Chemical Engineering, Shanghai Jiao Tong University, Shanghai, 200240, China.

[#]These authors contribute equally to this work.

[*]Correspondences: Liang Zhang: liangzhang2014@sjtu.edu.cn (Lead Contact); Jianxiu Yu: Jianxiu.Yu@gmail.com; Hao Wang: wang.hao@zs-hospital.sh.cn

This PDF file includes:

Supplementary Tables 1-5;

Supplementary Figures 1-27.

Supplementary Tables

Supplementary Table 1. Characteristics of 47 cases of lung adenocarcinoma specimens in a Chinese cohort. pT refers to pathological tumor size; pN refers to pathological lymph nodes; M refers to metastasis; pTNM stage refers to the combined evaluation of pT, pN, and M.

Patient ID	Age at diagnosis (year)	Gender	Tumor size (cm)	pT	pN	M	pTNM stage
1	50	Male	2	1b	0	0	IA
2	51	Female	1.5	1b	0	0	IA
3	69	Female	3	1c	1	0	IIB
4	64	Male	1.8	1b	0	0	IA
5	70	Male	1.8	1b	0	0	IA
6	75	Female	2.2	1c	0	0	IA
7	60	Female	0.9	1a	0	0	IA
8	76	Female	1.8	1b	0	0	IA
9	58	Male	1.5	1b	0	0	IA
10	65	Female	3.2	2a	0	0	IB
11	64	Female	2.2	1c	0	0	IA
12	67	Male	1.2	1b	0	0	IA
13	72	Male	2	1b	0	0	IA
14	48	Male	2.5	1c	0	0	IA
15	46	Female	2	1b	0	0	IA
16	78	Female	2.5	1c	0	0	IA
17	71	Female	2.2	1c	0	0	IA
18	64	Female	2	1b	0	0	IA
19	67	Female	3	1c	0	0	IA
20	61	Male	2.5	1c	0	0	IA
21	69	Male	3.5	2a	0	0	IB
22	39	Female	1.4	1b	0	0	IA
23	65	Female	1.2	1b	0	0	IA
24	52	Female	2.2	1c	0	0	IA
25	44	Male	1.4	1b	0	0	IA
26	50	Female	1.8	1b	0	0	IA
27	57	Female	3.4	2a	0	0	IB
28	59	Female	4	2a	2	0	IIIA
29	66	Female	2.5	1c	0	0	IA
30	64	Male	2	1b	2	0	IIIA
31	62	Female	2.8	1c	0	0	IA
32	49	Male	1	1a	0	0	IA
33	69	Female	1.5	2a	0	0	IB

Patient ID	Age at diagnosis (year)	Gender	Tumor size (cm)	pT	pN	M	pTNM stage
34	58	Male	1.7	1b	0	0	IA
35	36	Female	0.9	1a	0	0	IA
36	47	Female	1	1a	0	0	IA
37	50	Female	1.8	1b	0	0	IA
38	67	Male	4.1	2b	1	0	IIB
39	60	Male	3	1c	0	0	IA
40	57	Male	2	1b	0	0	IA
41	67	Female	2	1b	0	0	IA
42	61	Male	2.5	1c	0	0	IA
43	66	Male	3.5	2a	0	0	IB
44	76	Male	2.5	1c	0	0	IA
45	78	Female	3.5	2a	0	0	IB
46	40	Male	2.2	2a	0	0	IB
47	65	Female	2.8	1c	0	0	IA

Supplementary Table 2. Primers used in qPCR assays and knockdown assays.

qPCR primers	
PRPS1-F	5'-GAGACCTGTGTGGAAATTGGTGA-3'
PRPS1-R	5'-TTGTCATTGATTTCGCCACAACC-3'
PRPS2-F	5'-TGCCTGCAAGATTGCGTCAT-3'
PRPS2-R	5'-AAATTGGGGCACGACTCTCT-3'
METTL16-F	5'-TGGTATTTCCTCGCAACAGA-3'
METTL16-R	5'-AAGAGCATCCATCAGGAGTG-3'
METTL3-F	5'-AGCCTTCTGAACCAACAGTCC-3'
METTL3-R	5'-CCGACCTCGAGAGCGAAAT-3'
METTL14-F	5'-AGTGCCGACAGCATTGGTG-3'
METTL14-R	5'-GGAGCAGAGGTATCATAGGAAGC-3'
β-actin-F	5'-GGACTTCGAGCAAGAGATGG-3'
β-actin-R	5'-AGCACTGTGTTGGCGTACAG-3'
shRNA for knock down assays	
PRPS2-S1-F	5'-CCGGTTGCAGTGCTTGTATTGGTTTAACTCGAGTTAAACCAATACAAGCACTGCATTTTTG-3'
PRPS2-S1-R	5'-AATTCAAAAATGCAGTGCTTGTATTGGTTTAACTCGAGTTAAACCAATACAAGCACTGCAA-3'
PRPS2-S2-F	5'-CCGGTCCATACGCCCGACAAGATAAACTCGAGTTTATCTTGTCGGGCGTATGGTTTTTG-3'
PRPS2-S2-R	5'-AATTCAAAAACCATACGCCCGACAAGATAAACTCGAGTTTATCTTGTCGGGCGTATGGA-3'
PRPS2-S3-F	5'-CCGGTGTCACAAACACAATTCCGCAACTCGAGTTGCGGAATTGTGTTTGTGACTTTTTG-3'
PRPS2-S3-R	5'-AATTCAAAAAGTCACAAACACAATTCCGCAACTCGAGTTGCGGAATTGTGTTTGTGACA-3'
siRNA for knock down assays	
siMETTL16-1	5'-GGAAUUAUCCCUCAAAGCATT-3'
siMETTL16-2	5'-CCCAAAGUAACGUACACUGAATT-3'
siMELLT3-1	5'-CUGCAAGUAUGUUCACUAUGATT-3'
siMETTL3-2	5'-GGUUGGUGUCAAGGAAAUTT-3'
siMETTL14-1	5'-GGAUGAAGGAGAGACAGAUTT-3'
siMETTL14-2	5'-AAGGAUGAGUUAAUAGCUAAATT-3'

Supplementary Table 3. Kinetic characterization of PRPS1, PRPS2, and various chimeric and mutated enzymes in catalyzing either ATP or R5P by using an MK/LDH/PK coupled assay.

Enzyme	K_m (μM)		K_{cat} (s^{-1})		K_{cat}/K_m ($\mu M^{-1}s^{-1}$)	
	ATP	R5P	ATP	R5P	ATP	R5P
PRPS1	175.6 ± 7.4	55.0 ± 2.6	3512	2560	19.9	46.6
PRPS2	317.5 ± 17.6	36.6 ± 2.7	233	134	0.7	3.6
PRPS1(+3AA)	268.5 ± 5.2	97.4 ± 2.4	1177	966	4.4	10.2
PRPS2(-3AA)	374.2 ± 8.0	115.3 ± 4.8	1231	1091	3.3	9.4
PRPS1-ΔC	36±3.3	7.05±0.8	1.98	1.58	0.055	0.225
PRPS2-ΔC	19.43±2.9	1.84±0.87	1.58	1.46	0.08	0.79
PRPS1(+3AA/K153Q)	339.5 ± 28.7	55.0 ± 2.6	261	1003	0.77	18
PRPS2(-3AA/Q156K)	359.8 ± 16.5	87.1 ± 4.2	4414	3333	12.26	38.27
PRPS1(R96A)	N/A	N/A	N/A	N/A	N/A	N/A

Supplementary Table 4. Statistics of diffraction data and structure refinement.

	PRPS2	PRPS1-GDP	PRPS1(+3AA)
**Data collection**			
Space group	$P2_1$	$P6_5$	$P4_3$
Cell dimensions			
a, *b*, *c*(Å)	94.70, 73.60, 170.50	186.88, 186.88, 160.64	108.08, 108.08, 659.67
α, β, γ (°)	90.00, 94.44, 90.00	90.00, 90.00, 120.00	90.00, 90.00, 90.00
Wavelength (Å)	0.9873	0.9873	0.9873
Resolution (Å)*	50-2.75 (2.80-2.75)	50-3.0 (3.11-3.00)	50-3.10 (3.21-3.10)
R_{pim} (%)	7.2(37.0)	6.8(31.6)	15.3(52.9)
$I/\sigma I$	9.6(1.8)	11.2(2.6)	6.2 (1.7)
Completeness (%)	94.1(71.7)	100(100)	100(100)
Redundancy	4.2(3.0)	17.4(12.0)	13.2(9.3)
CC(1/2) (%)**	68.9	76.4	64.5
**Refinement**			
Resolution (Å)	46.90-2.74	48.70-3.00	50.00- 3.10
No.reflections	54437	62270	133985
R_{work}/R_{free} (%)	27.3/28.6	24.7/29.6	19.6/25.0
No.atoms			
Protein	13730	19598	42184
Water	194	65	N/A
Ligand/ion	301	450	13
B-factors			
Protein	48.76	55.87	56.54
Water	35.90	40.06	N/A
Ligand/ion	81.75	66.93	101.87
R.m.s deviations			
Bond lengths (Å)	0.03	0.02	0.01
Bond angles (°)	2.35	1.84	1.71
Ramachandran plot (%)#			
Favored	94.1	90.4	95.9
Allowed	5.4	8	3.9
Outliers	0.5	1.6	0.2

Rotamer outliers (%)	8.1	2.2	4.4
Clash scores	27	11	15

* Highest-resolution shell is shown in parentheses; **Data shown in highest-resolution shell; #The values were calculated by using Procheck from CCP4 software package.

Supplementary Table 5. Flag-tag based high-affinity pulldown results from Flag-tagged PRPS2 or PRPS1 ectopically expressed H1299 and HEK293T stable cell lines.

Names	Total	Elements
293A & 293B & HA & HB	38	ACTB; RPS2; KRT14; EEF2; KRT9; PRPSAP2; DDX39A; TCP1; RACK1; PKM; PUF60; RPL22; KRT1; RPS20; KRT10; TUBB4B; EIF5A; PRPS1; MIF; KRT5; SLC25A5; ATP5F1A; HSPD1; RPL12; HSPA5; TUBB; CFL1; CCT2; NPM1; PRPS2; PRPSAP1; CCT8; RPS14; HSP90AB1; NCL; RAN; PRSS1; KRT2;
293A & HA & HB	6	TXN; KRT74; RPL3; CLTC; RPSA; YWHAZ;
293B & HA & HB	25	ENO1; SLC25A3; KRT77; KRT76; ATP5F1B; PCNA; AHSA1; RPL11; RANBP1; TUBA8; KRT13; DSTN; YWHAE; TUBB6; RPS12; KRT16; RPS3; KRT79; PRDX1; ABCF2; HSPA8; TUBB4A; UBC; HSP90AA1; HRNR;
293A & 293B & HB	5	AASDHPPT; C1QBP; PCBP1; RPS15A; LDHB
HA & HB	33	RPS11; ANXA2; CNBP; XP32; MTHFD1; HEL-S-39; PARK7; RPS5; SFPQ; HIST2H3A; PTI-1; PFN1; LGALS1; YWHAQ; SRRT; CKB; EIF1AX; GOLPH3; ARF4; SF3B1; OTUB2; GAGE2B; RPS16; ATP5IF1; RPL9; IGKV2D-29; PSMD4; GARS; SF1; HBA2; MAGEA4; TRA1; RPS27;
293A & HA	5	DDB1; HSDL2; DCAF16; MTFR1L; PTBP1;
293B & HA	2	PPA2; ATP5PO;
293A & HB	1	CTPS1;
293B & HB	7	VIM; EIF2S1; RAB6B; PCBP2; MCM7; TECR; **MAT2A**;
293A & 293B	40	CSTA; H1-3; HDDC2; PDHA1; RPS18; HNRNPH1; RPL7; KRT82; PCBP3; HSPA1B; TUBB2B; LYZ; HSPA2; PRPS1L1; HNRNPA2B1; PHGDH; HNRNPK; HSPA9; RPL23A; KRT6B; ALB; GAPDH; SLC25A11; UBA52; TUFM; (H4-16; H4C1; H4C11; H4C12; H4C13; H4C14; H4C15; H4C2; H4C3; H4C4; H4C5; H4C6; H4C8; H4C9); S100A8; RPL38; PRDX4; CCT3; RPL18; CLPX; SDHA; EEF1G; EEF1A1; RPS8; TUBA1B; FHL1; RPL4; SUCLG1

Supplementary Figures

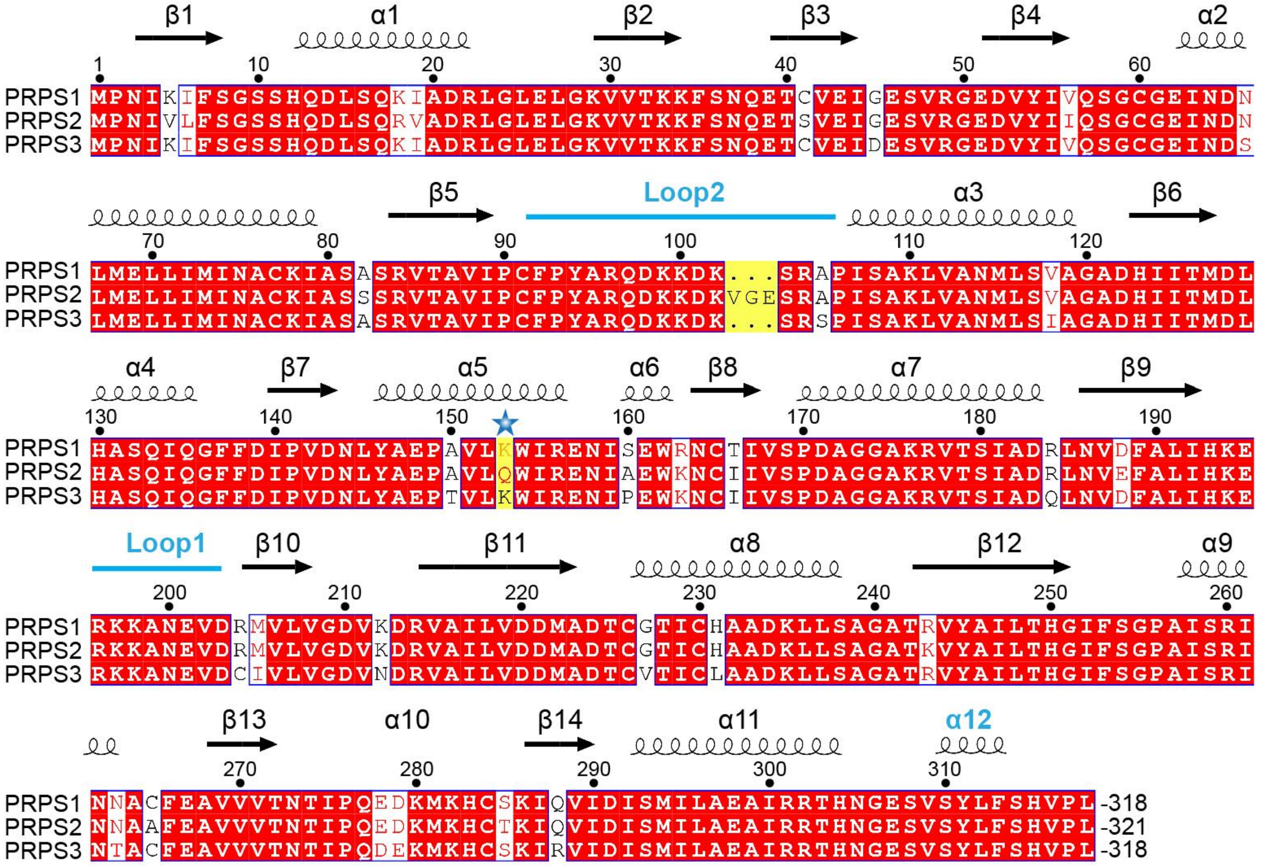

Supplementary Figure 1. Multiple structural alignments of human PRPS homologs (PRPS1, PRPS2, and PRPS3). The structural alignment was generated using the Espript online server (http://espript.ibcp.fr/ESPript/ESPript/). Identical residues among the three proteins were shaded in red. The three non-conserved amino acids (V103, G104, E105) found only in PRPS2 were highlighted in yellow, while the key secondary elements Loop1 and Loop2 were labeled in cyan. The other key non-conserved residue, K153 in PRPS1 (colored in orange), and the corresponding residue, Q156 in PRPS2 (colored in red), were marked with a blue star.

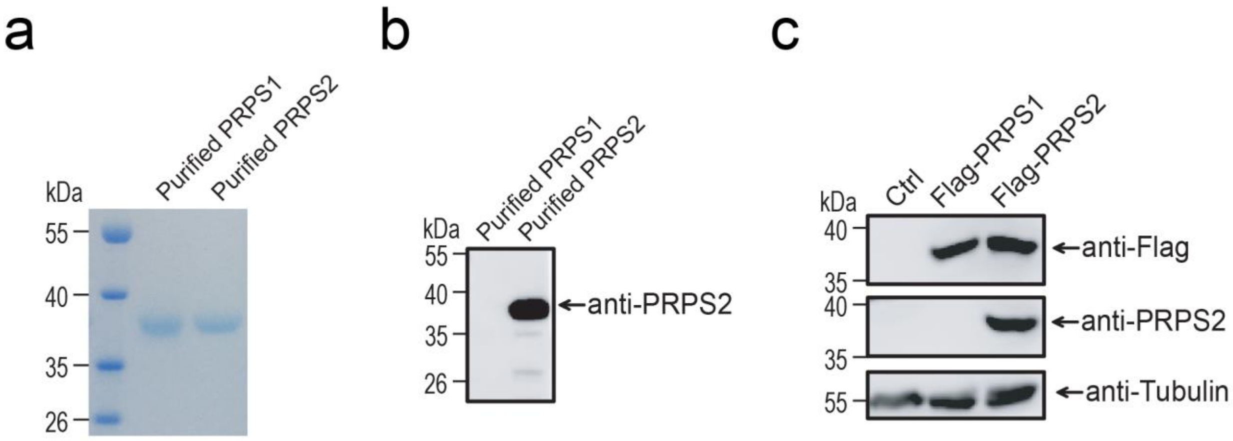

Supplementary Figure 2. PRPS2-specific antibody generation and evaluation. (a) Recombinant PRPS1 and PRPS2 were expressed and purified from *E. coli* system. (b) The PRPS2 antibody differentiates PRPS2 from PRPS1 against recombinant PRPS2 and PRPS1 purified from *E. coli* system. (c) PRPS2 antibody differentiates PRPS2 from PRPS1 against purified, ectopically expressed, and flag tagged PRPS2 and PRPS1 from HEK293FT mammalian cells. Anti-Flag antibody and anti-PRPS2 antibody were used in the assay.

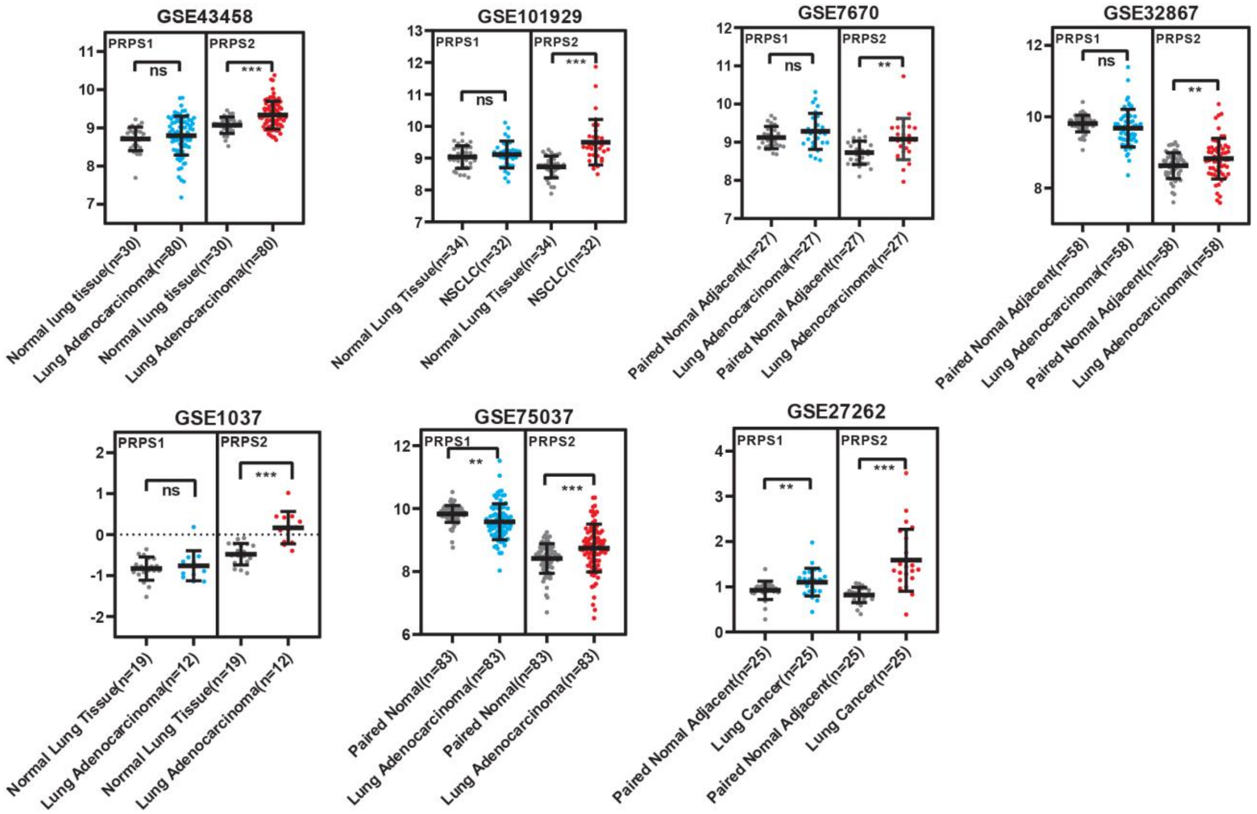

Supplementary Figure 3. Endogenous mRNA expression levels of the *PRPS2* or *PRPS1* gene in clinical lung adenocarcinoma specimen tissues (LUAD) compared to the paired normal tissues from the GEO database.

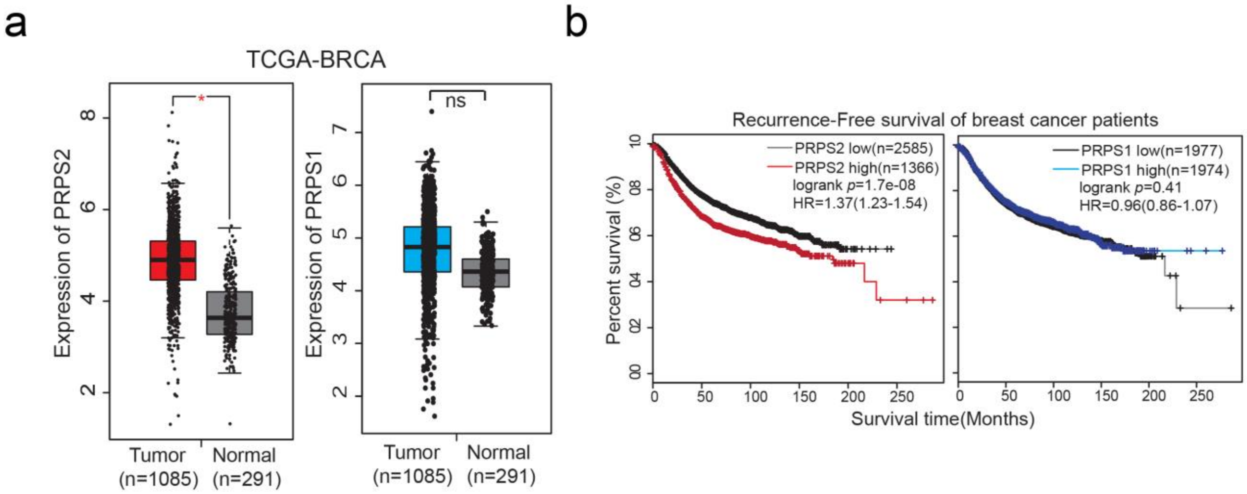

Supplementary Figure 4. (a) Endogenous mRNA expression levels of the *PRPS2* or *PRPS1* gene in clinical breast cancer specimen tissues (BRCA) compared to the paired normal tissues from the TCGA database. (b) The effect of *PRPS1* or *PRPS2* expression on Kaplan-Meier survival curves of breast cancer patients indicated that high expression of PRPS2, but not PRPS1, is associated with poorer patient prognosis. Statistical analysis was based on log-rank tests, and the p-value is indicated.

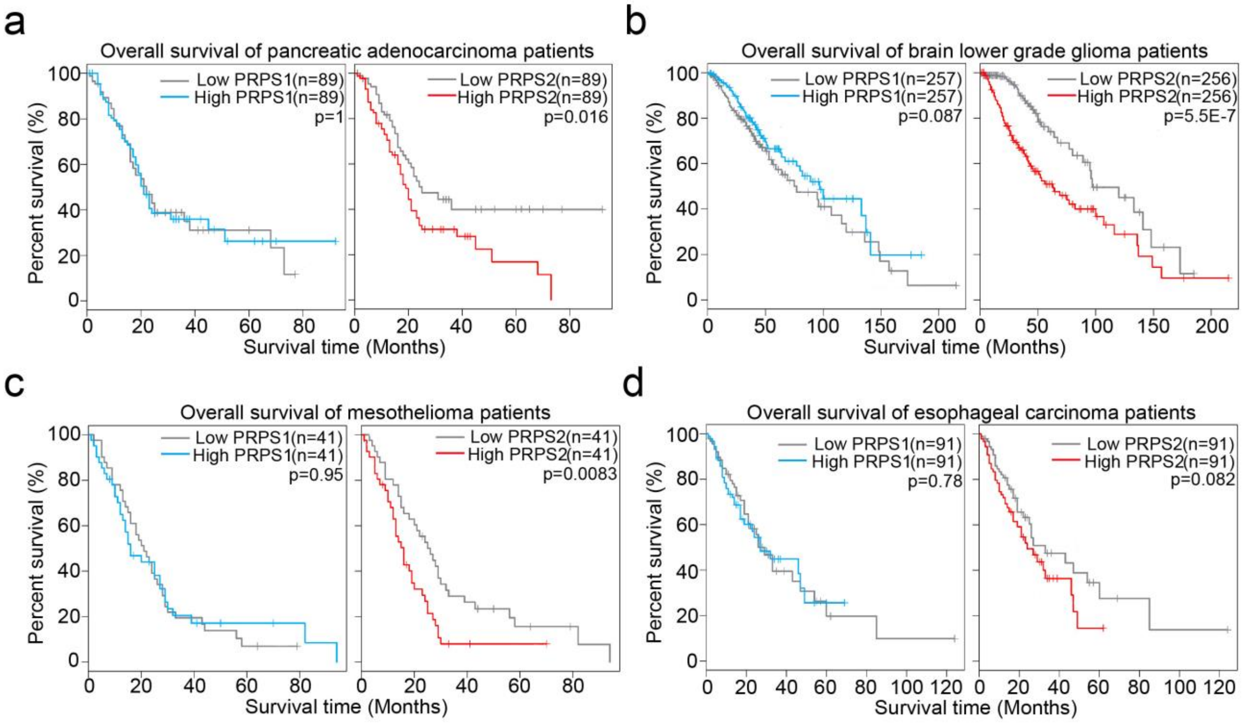

Supplementary Figure 5. High expression of PRPS2, but not PRPS1, is associated with poorer patient prognosis in pancreatic adenocarcinoma (a), brain lower-grade glioma cancer (b), mesothelioma (c), and esophageal carcinoma cancer patients (d). Statistical analysis was performed using the log-rank test and indicated as p-value.

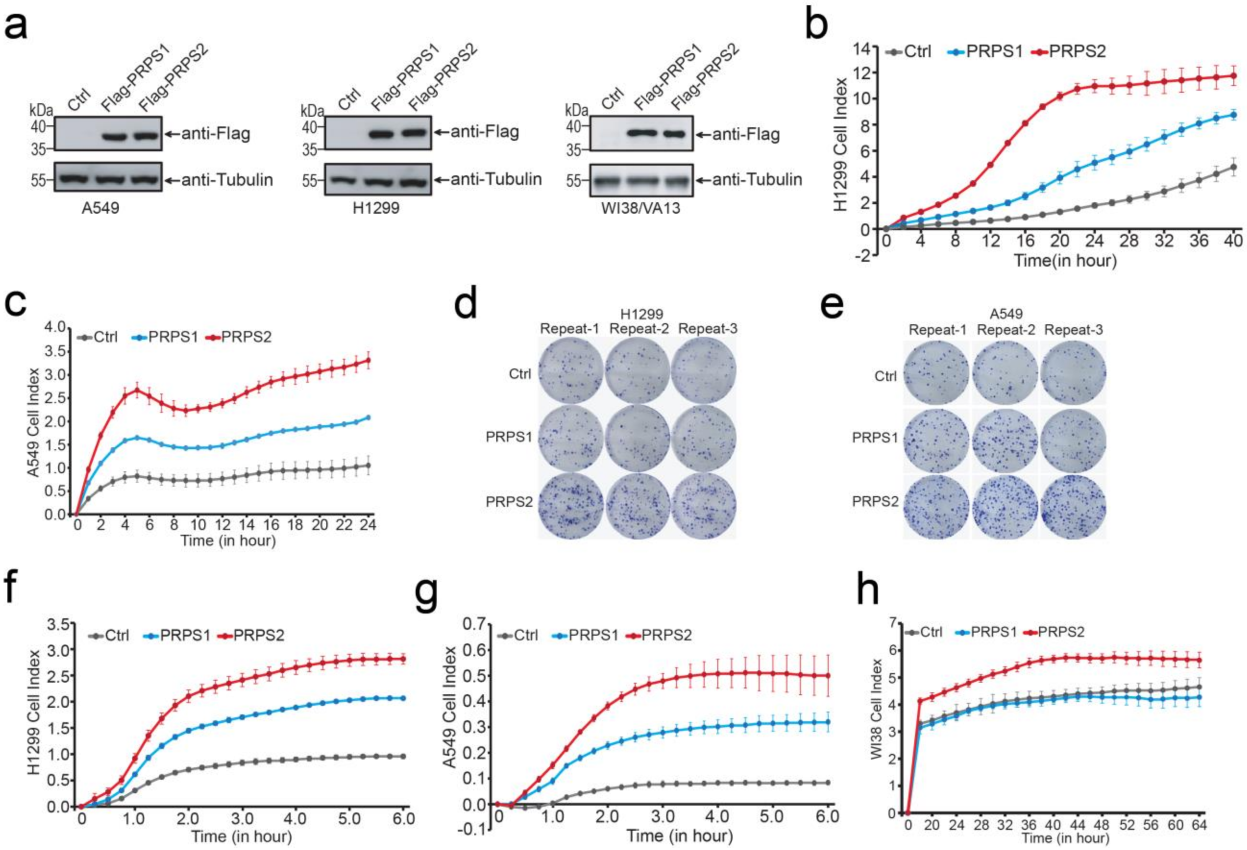

Supplementary Figure 6. Cell proliferation and migration of PRPS2 or PRPS1 ectopically overexpressed H1299 or A549 cell lines. (a) Western blot evaluation of ectopic overexpression of PRPS2 or PRPS1 in H1299, A549, and human lung normal fibroblast cell WI38/VA13 cell lines. (b, c) Cell proliferation of PRPS2 or PRPS1 ectopically overexpressed H1299 (b) and A549 cell lines (c). (d, e) Colony-formation assay on PRPS2 or PRPS1 ectopically overexpressed H1299 (d) and A549 (e) cell lines. (f, g) Cell migration of PRPS1 or PRPS2 ectopically overexpressed H1299 (f) and A549 (g) cell lines. (h) Cell proliferation of PRPS1 or PRPS2 ectopic overexpressed human lung normal fibroblast cell WI38/VA13 cell line. The data in (b-c) and (f-h) were plotted as the mean ± SDs of biological triplicates.

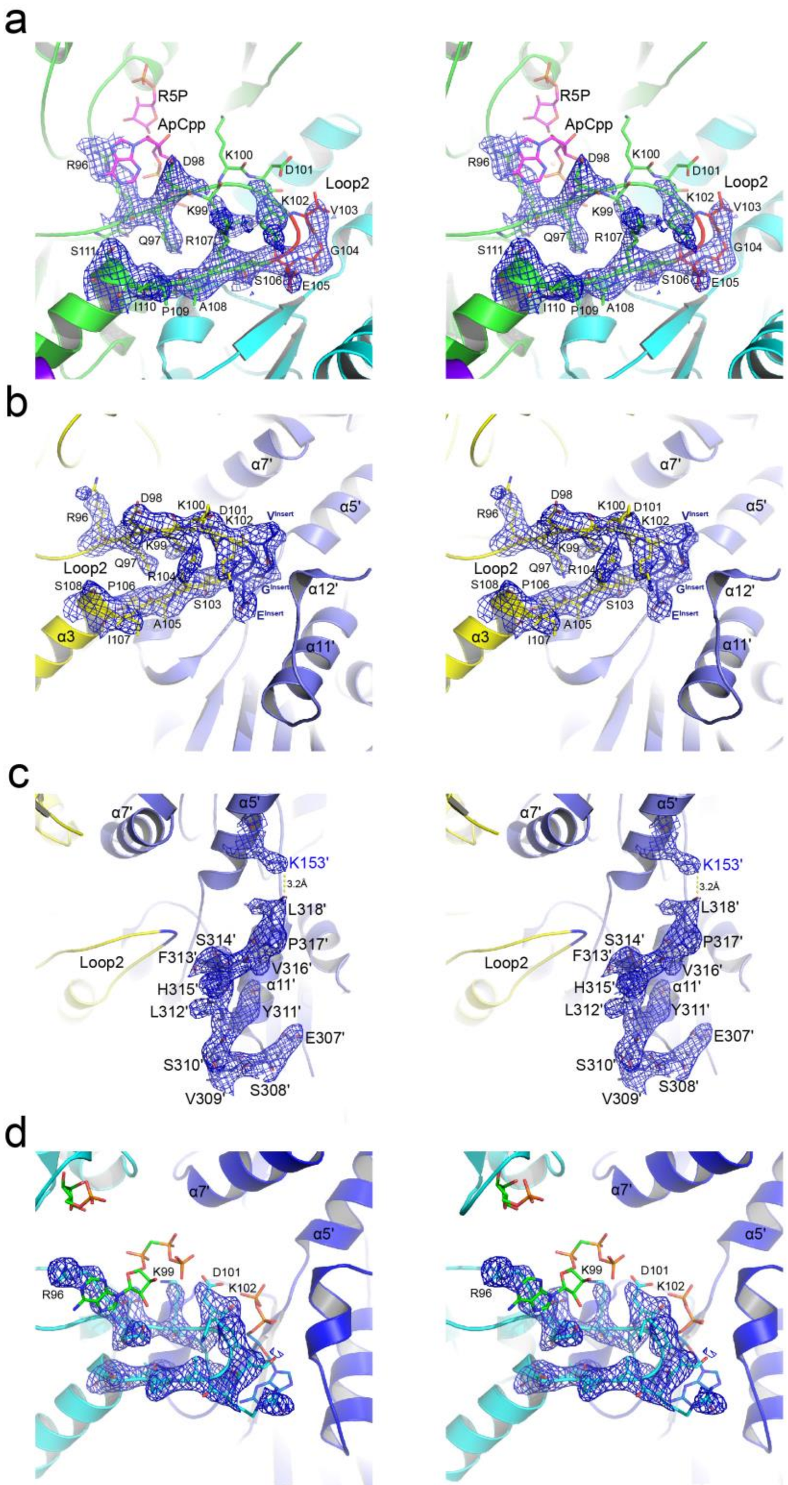

Supplementary Figure 7. The omit electron density maps around the PRPSs Loop2 and α12 helix regions. The Fo-Fc electron density maps are contoured at 2.5σ around PRPS2 Loop2 (a), PRPS1^{+3AA} Loop2 (b), PRPS1^{+3AA} C-terminal α12 helix and Lys153' (c), and PRPS1 Loop2 (d).

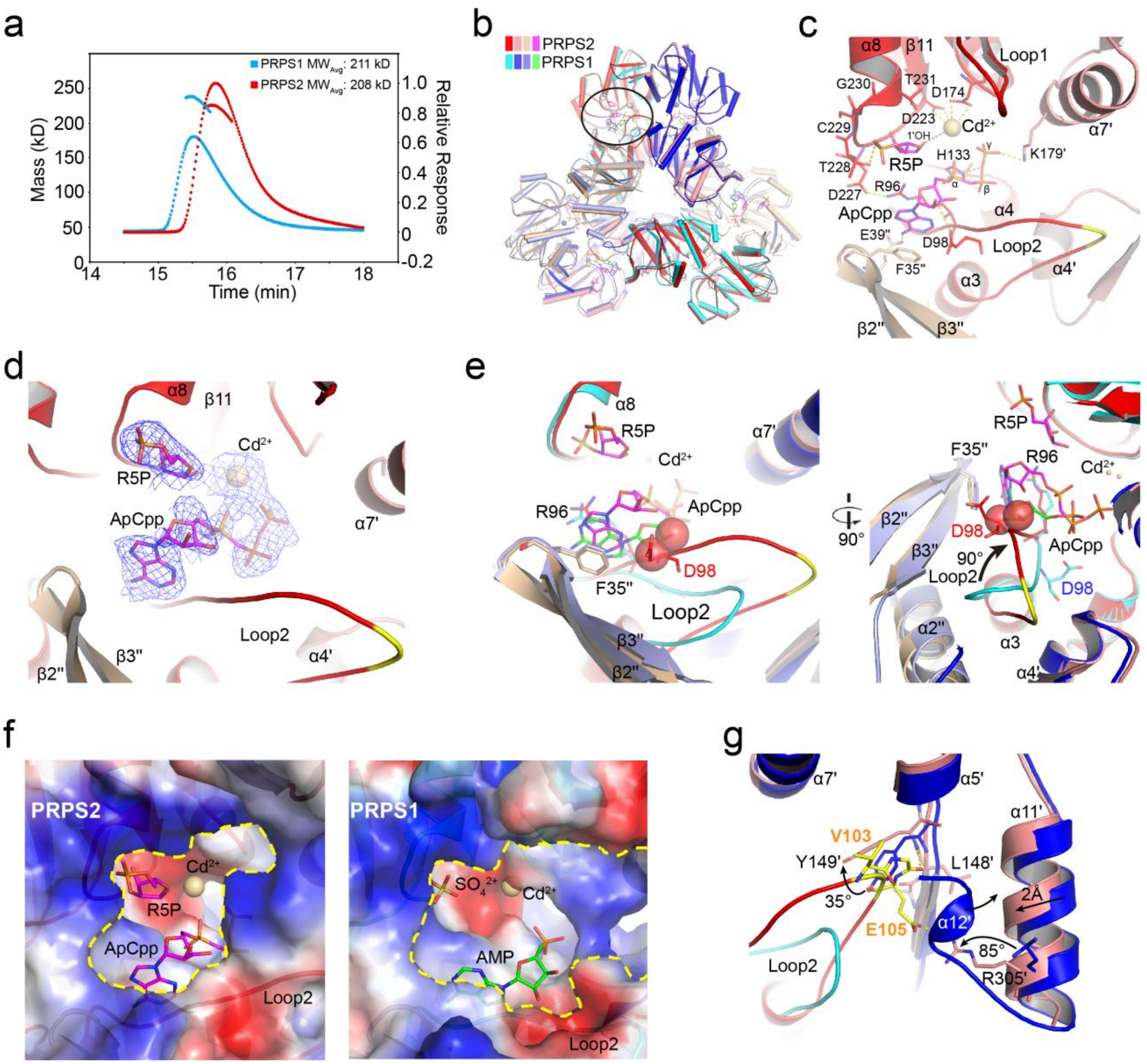

Supplementary Figure 8. Structural analysis of PRPS2. (a) Size-exclusion chromatography (SEC) coupled with multi-angle light scattering (MALS) analysis. The PRPS2 protein or PRPS1 was separated using a Wyatt Technology WTC-030S5 column. The corresponding average molecular weights of the fraction peaks were evaluated and analyzed using the Wyatt Technology SEC-MALS system. The calculated molecular masses of PRPS1 and PRPS2 throughout the peaks were labeled, and the molecular mass scaling was shown on the left-hand Y-axes. The chromatogram displays the readings of the laser detector, and the relative response scaling was shown on the right-hand Y-axes. The SEC-MALS analysis demonstrated that they exhibited a similar hexamer oligomerization in solution, confirming their high homology. (b) Structural superposition of PRPS2 and PRPS1 hexamer structures. The black circles indicate the

catalytic pockets of PRPS2 and PRPS1. (c) The binding of R5P, ApCpp, and Cd^{2+} inside the catalytic pocket of PRPS2. Yellow dashes indicate hydrogen bonds, and the gray dash indicates a ~5 Å distance between the 1'OH group of R5P and Cd^{2+}. The three key residues (3AA) present in PRPS2 Loop2 are colored in yellow. (d) The 2Fo-Fc electron density map contoured at 0.8σ around R5P, ApCpp, and Cd^{2+} ligands in the catalytic pocket of the PRPS2 structure. The electron density is shown as blue meshes. (e) Superposition of the PRPS2 and PRPS1 catalytic pocket regions. The longer Loop2 of PRPS2 adopts a ~90° perpendicular conformation compared to that of PRPS1, constraining the position of ApCpp and limiting its access. (f) The electrostatic surfaces of the PRPS2 and PRPS1 catalytic pocket entrances generated by PyMOL. The extreme ranges of red (negative) to blue (positive) in PRPS2 and PRPS1 represent the surface electrostatic potentials of -66.69 e/kT to +66.69 e/kT and -73.67 e/kT to +73.67 e/kT, respectively. The entrance edges are outlined with yellow dashes. (g) Superposition of the Loop2 and α12' helix regions of PRPS2 and PRPS1. The 3AA induces steric clashes with the α12' helix, causing ~35° or ~85° sidechain swinging of PRPS2 Try149' or Arg305' compared to those of PRPS1, respectively, pushing the α12' helix outwards, decreasing its stability and leading to its structural disorder in the PRPS2 complex structure. The PRPS2 3AA on Loop2 is colored in yellow, and labeled as orange V103 and E105. The black arrows indicate the rotation directions of the residue sidechains or helix.

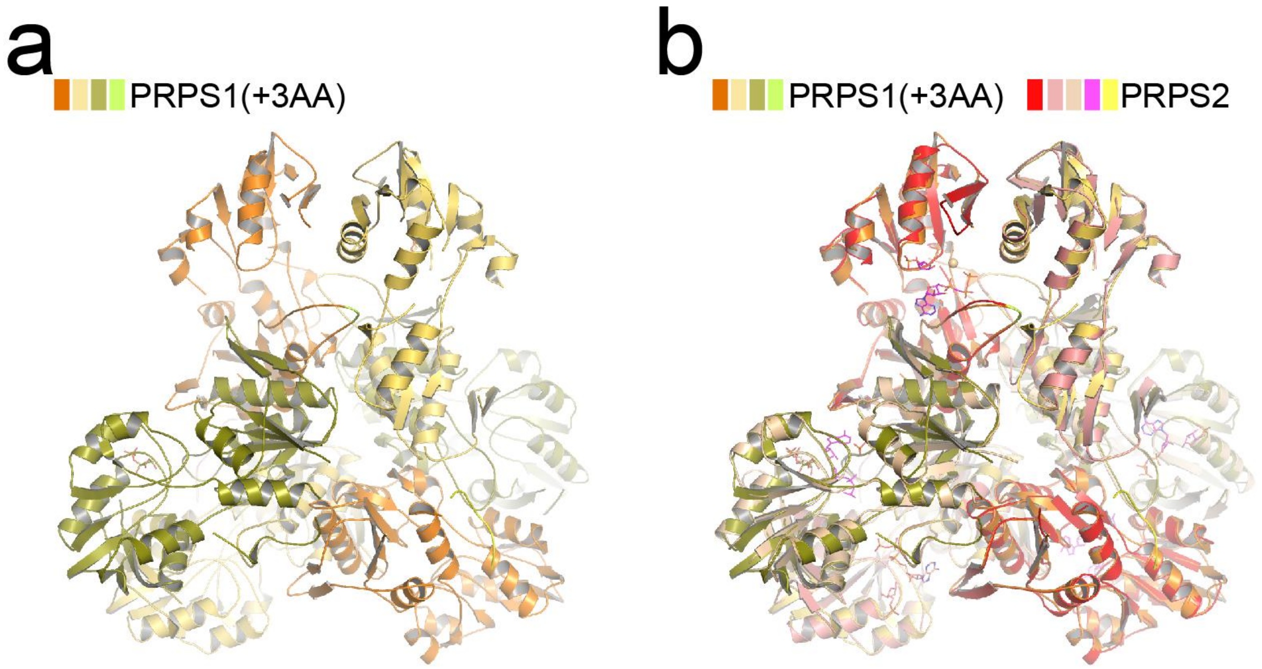

Supplementary Figure 9. Structural analysis of PRPS1(+3AA). (a) The overall structure of PRPS1(+3AA). (b) Superposition of the PRPS1(+3AA) and PRPS2 hexamer complex structures.

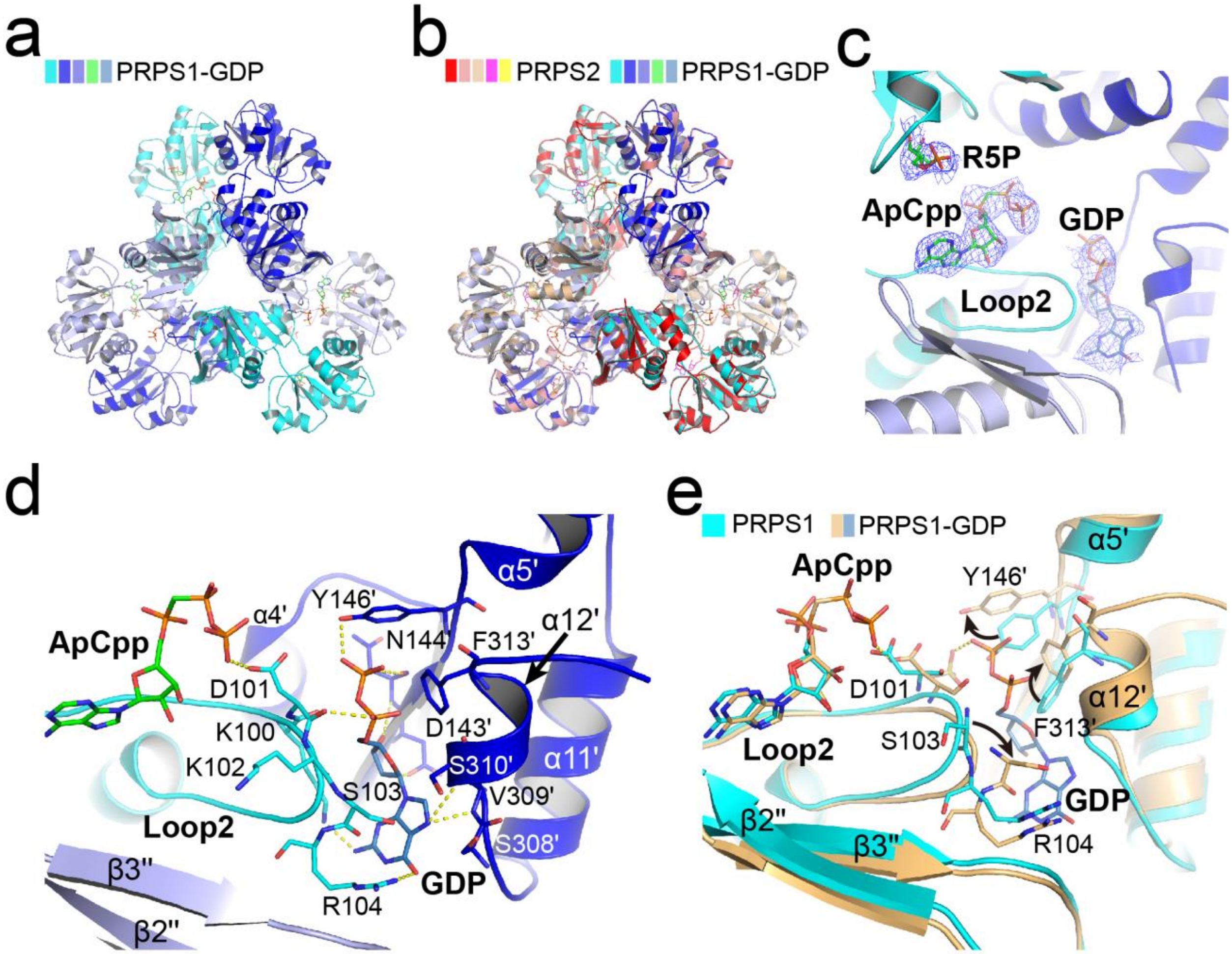

Supplementary Figure 10. Structural analysis of PRPS1 in complex with GDP. (a) Overall structure of the PRPS1-GDP complex. (b) Superposition of the PRPS1-GDP and PRPS2 hexamer complex structures. (c) 2Fo-Fc electron density map contoured at 1.0σ around R5P, ApCpp, and GDP (sky blue) ligands in the catalytic and allosteric sites of the PRPS1 structure. The electron density is shown as blue meshes. (d) The binding of GDP to the allosteric site of PRPS1. Yellow dashes indicate the H-bonds. (e) Superposition of the GDP binding site of PRPS1-GDP and PRPS1 indicates conformational changes of residues on Loop2, α12' and β7' upon GDP's binding. The black arrows indicate the residue sidechain rotations upon GDP's binding.

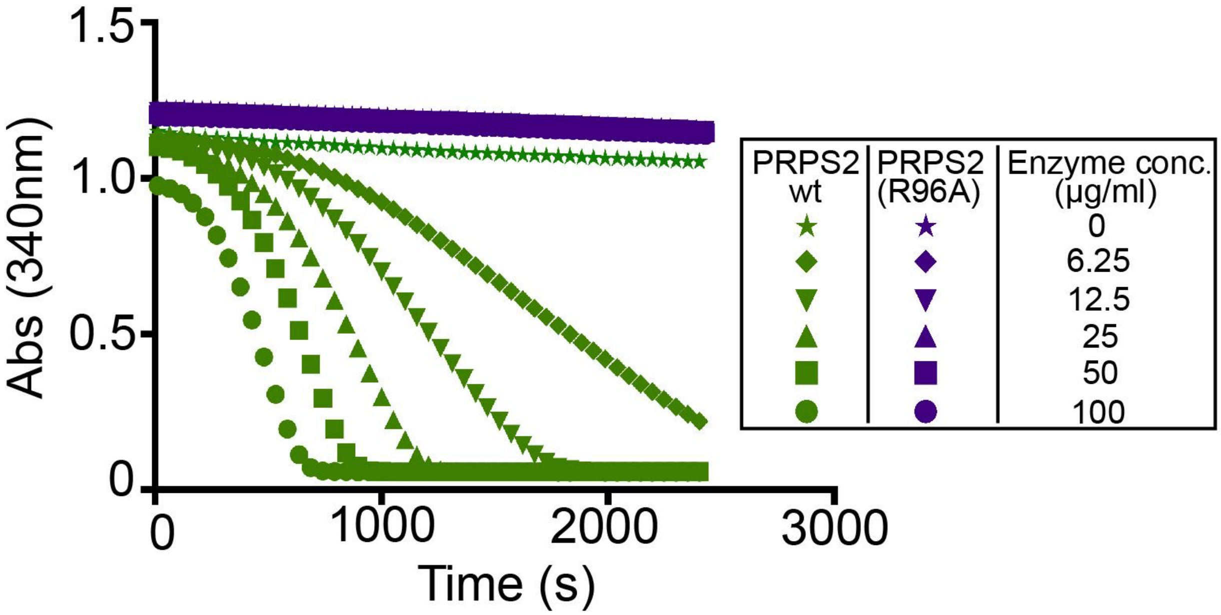

Supplementary Figure 11. Biochemical characterization of PRPS2(R96A). Enzymatic activity characterization of PRPS2 and PRPS2(R96A) indicated that R96A mutagenesis abolishes the enzymatic activity of PRPS2.

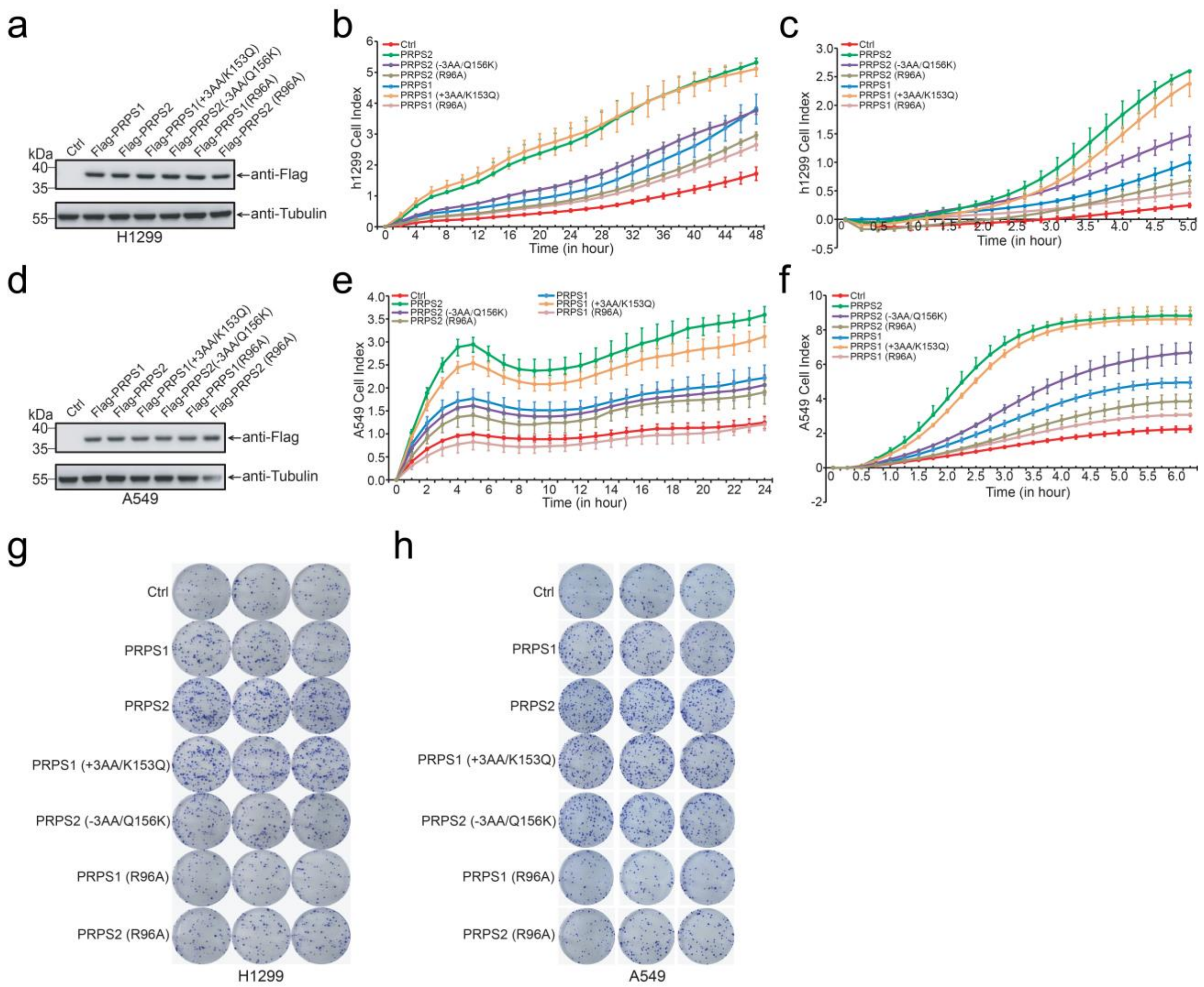

Supplementary Figure 12. Cell proliferation and migration of PRPS1, PRPS2, PRPS1(+3AA/K153Q), PRPS2(-3AA/Q156K), PRPS1(R96A) or PRPS2(R96A) ectopically expressed H1299 (a-c, g), and A549 (d-f, h) cell lines. Soft agar colony formation assay showed that ectopic expression of PRPS2 or PRPS1(+3AA/K153Q) promotes H1299 (g) and A549 (h) cell proliferation. The results were repeated by three times independently, and the corresponding statistical analysis results are shown in Figure 5. The data in (b-c) and (e-f) are plotted as the mean ± SDs of biological triplicates.

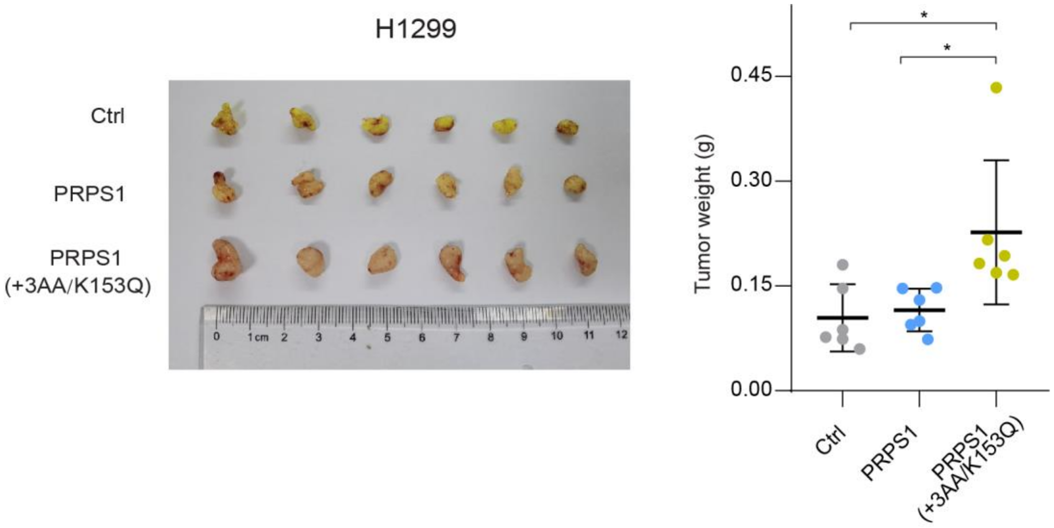

Supplementary Figure 13. The xenograft tumor growth assay by using H1299 cell line. The data in the right panel were plotted as the mean ± SDs of six biological replicates. *$p < 0.05$ for multiple comparisons calculated using one-way ANOVA with Tukey's HSD test.

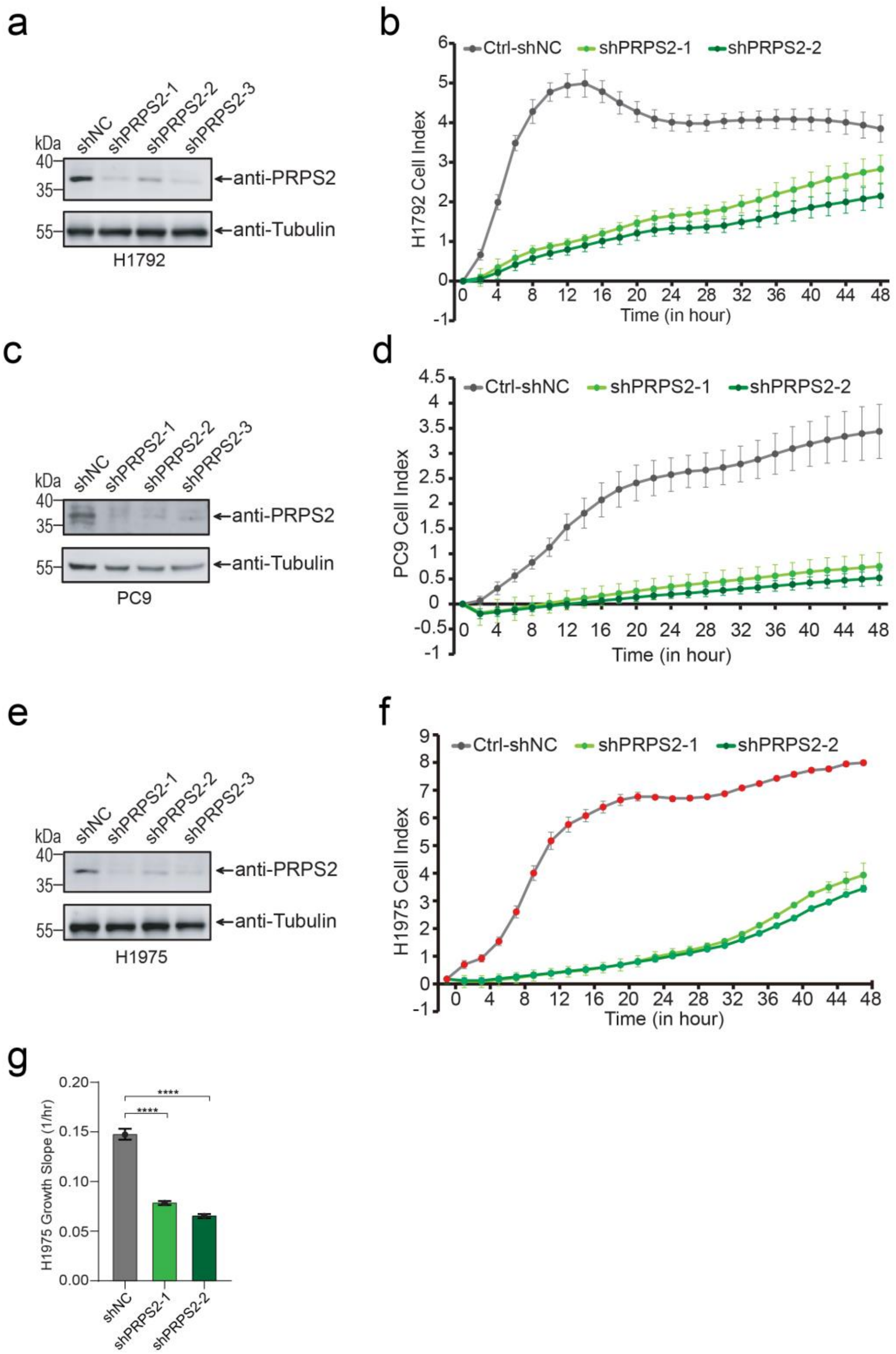

Supplementary Figure 14. Western blot analysis and cell proliferation of PRPS2 shRNA knockdown H1792 (a, b), PC-9 (c, d), and H1975 (e-g) cell lines. The data in (b), (d), (f), and (g) are plotted as the mean ± SDs of biological triplicates. ****p < 0.0001 for multiple comparisons calculated using one-way ANOVA with Tukey's HSD test.

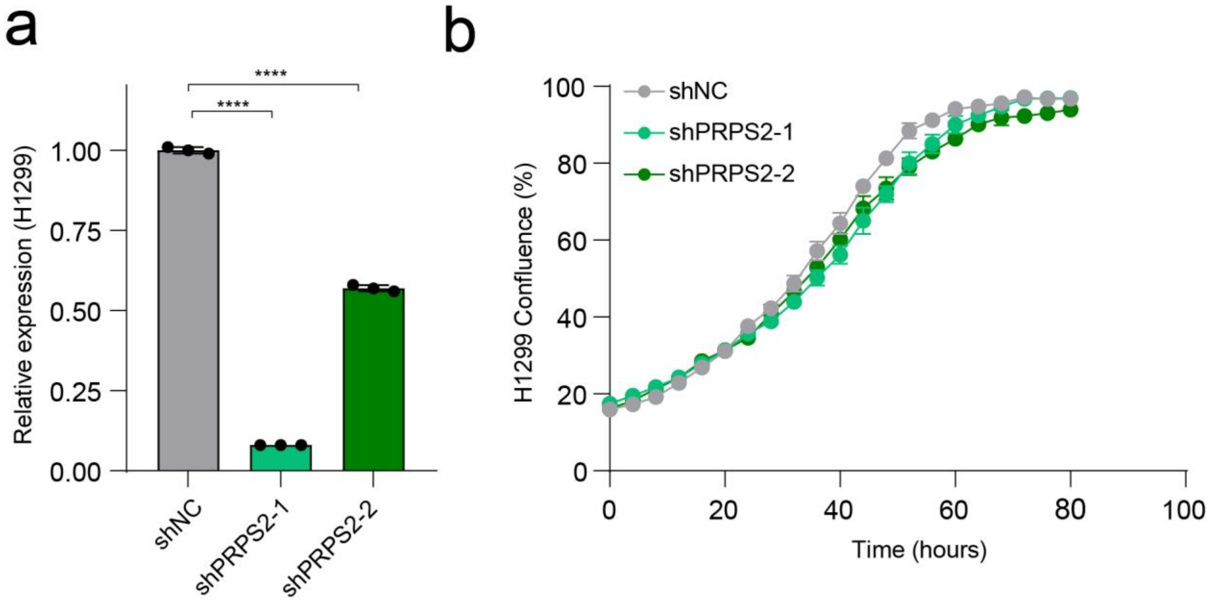

Supplementary Figure 15. The shRNA knockdown of PRPS2 in H1299 cell line with low endogenous PRPS2 expression level. The data in (a-b) were plotted as the mean ± SDs of biological triplicates. ****$p < 0.0001$ for multiple comparisons, calculated using one-way ANOVA with Tukey's HSD test.

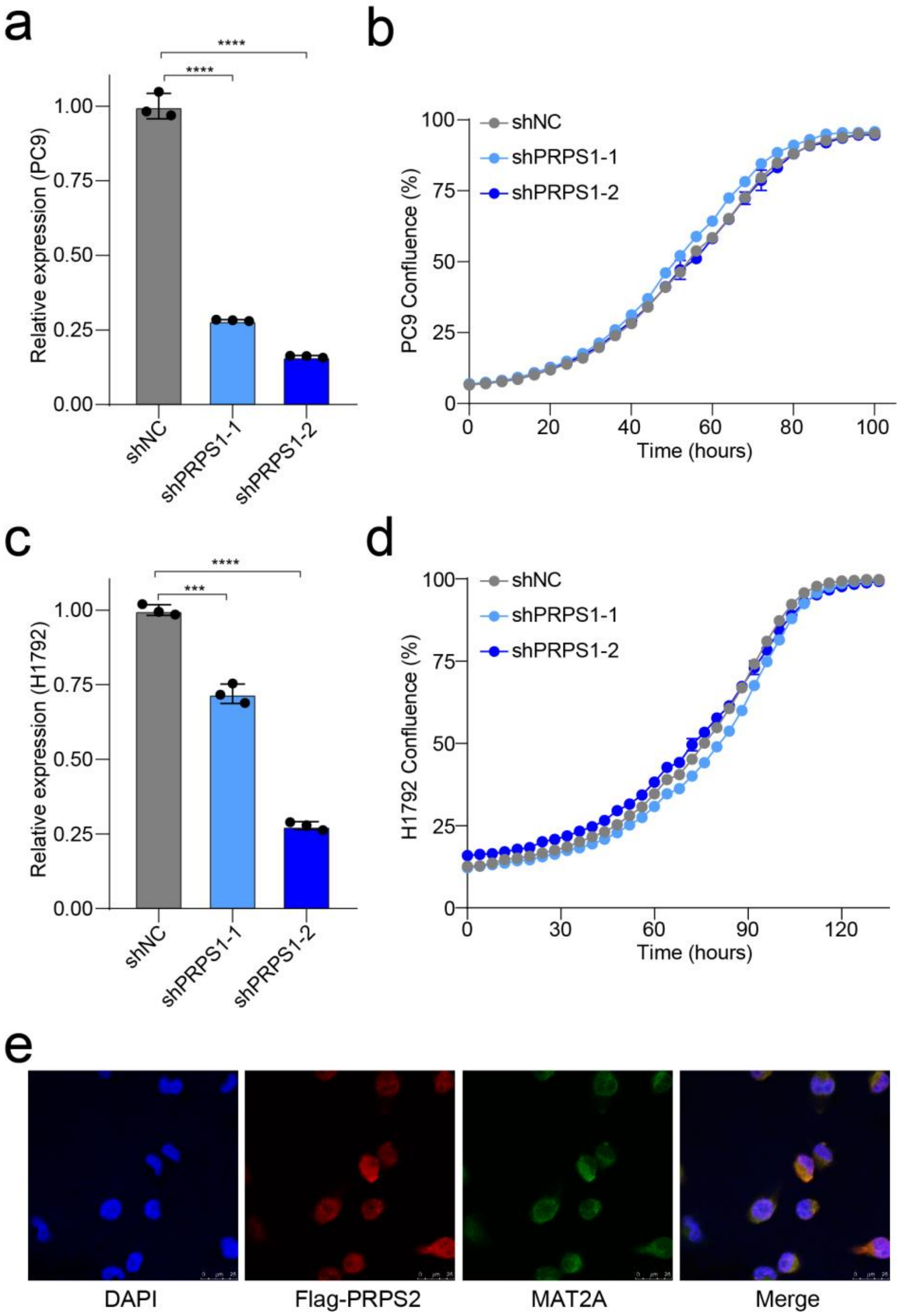

323 **Supplementary Figure 16. Cell proliferation of PRPS1 shRNA knockdown in H1792 and**
324 **PC9 cell lines, and location of PRPS2 and endogenous MAT2A in H1299 cells.** (a-d) qPCR
325 and cell proliferation of PRPS1 shRNA knockdown H1792 (a, b) and PC9 (c, d) cell lines by
326 using the IncuCyte system. (e) Immunofluorescence assay showing the location of PRPS2 and
327 endogenous MAT2A from H1299 cells transfected with FLAG-PRPS2. The data in (a-d) were
328 plotted as the mean ± SDs of biological triplicates. ***p < 0.0001, and ****p < 0.0001 for
329 multiple comparisons calculated using one-way ANOVA with Tukey's HSD test.

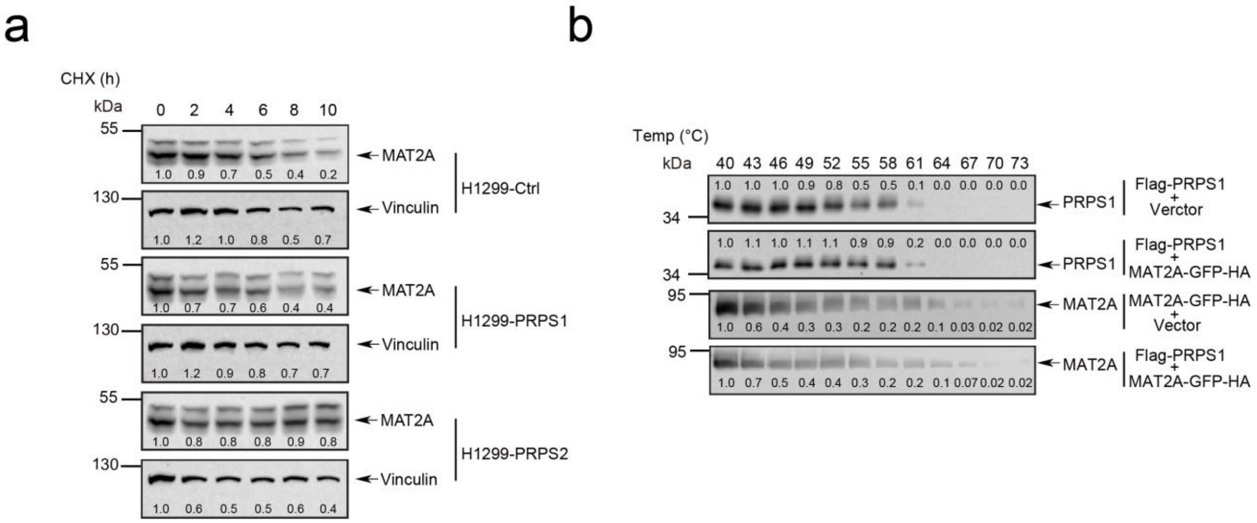

Supplementary Figure 17. CHX and cellular thermal shift assays. (a) MAT2A expression levels in PRPS1and PRPS2 ectopically expressed H1299 cells treated with cycloheximide (CHX). (b) The stability of MAT2A protein in a cellular thermal shift assay in HEK293T cells transfected with FLAG-PRPS1 and HA-MAT2A. The quantification values of the band intensity were labeled.

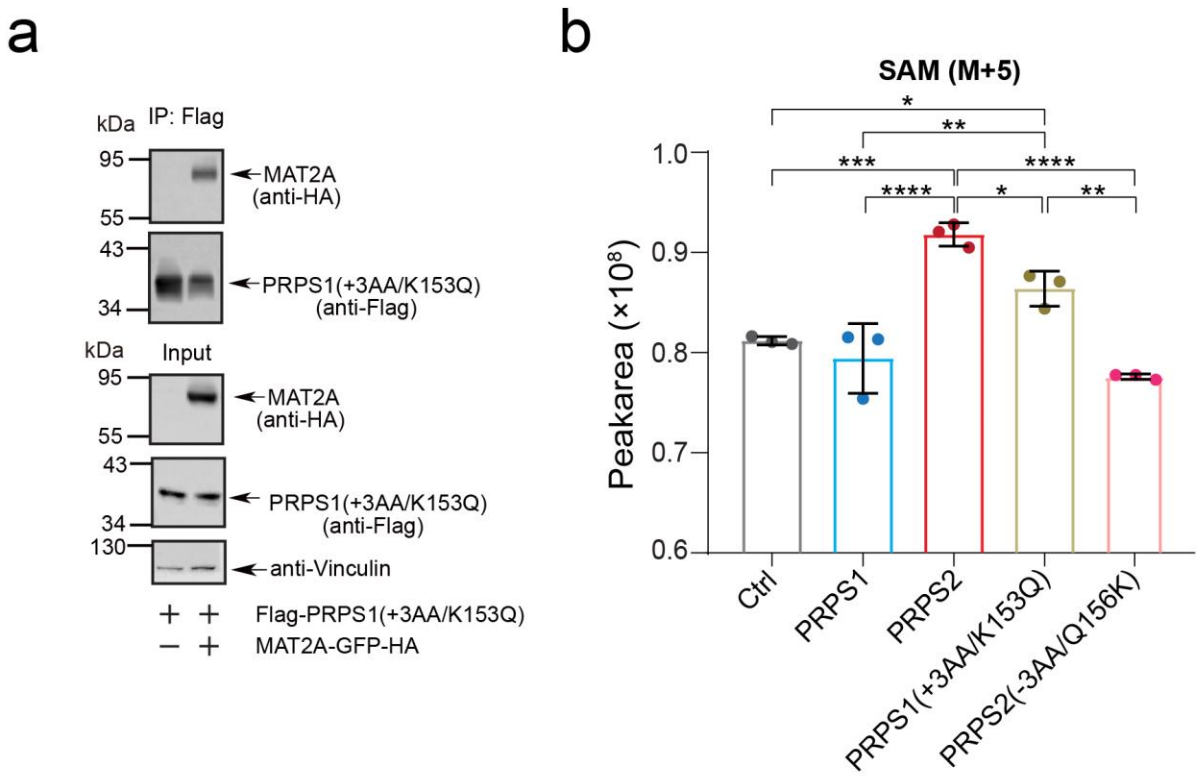

Supplementary Figure 18. PRPS1(+3AA/K153Q) interacts with MAT2A and stimulates SAM synthesis. (a) Immunoprecipitation and Western blot analysis demonstrated the interaction between PRPS1(+3AA/K153Q) and MAT2A in HEK293T cells transfected with FLAG-PRPS1(+3AA/K153Q) and HA-MAT2A. (b) Peak areas of ^{13}C-labeled SAM were measured by targeted LC-MS/MS in PRPSs ectopically expressed H1299 cells. The data were presented as the mean ± SDs of biological triplicates. *$p < 0.05$, **$p < 0.01$, ***$p < 0.001$, and ****$p < 0.0001$ for multiple comparisons calculated using one-way ANOVA with Tukey's HSD test.

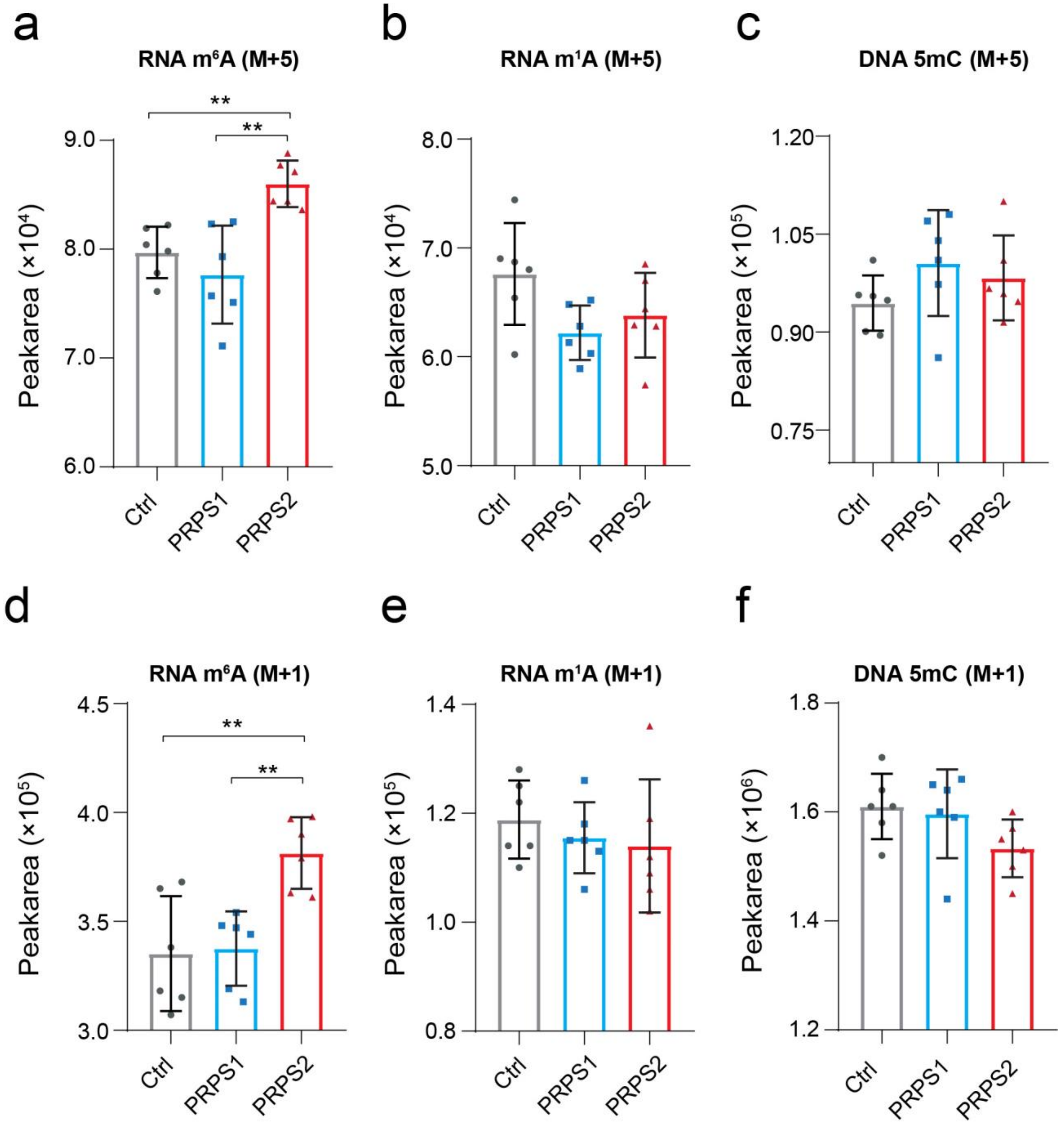

Supplementary Figure 19. Peak areas of ^{13}C-labeled methylated nucleotides from DNA 5mC, RNA m^{6}A, and RNA m^{1}A in PRPS2 or PRPS1 ectopically expressed A549 cell lines. (a-c)^{13}C stable isotope-labeled carbon flow from $^{13}C_6$-glucose. (d-f) ^{13}C stable isotope-labeled carbon flow from $^{13}C_1$- methionine. The data in (a-f) were plotted as the mean ± SDs of six biological replicates. **p < 0.01 for multiple comparisons calculated using one-way ANOVA with Tukey's HSD test.

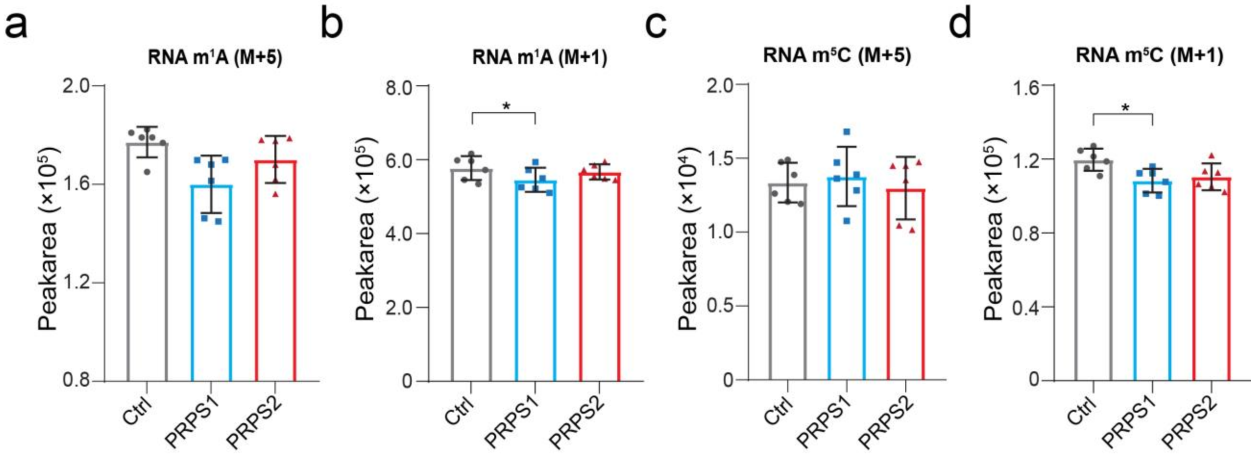

Supplementary Figure 20. Peak areas of ^{13}C-labeled ($^{13}C_6$-glucose or $^{13}C_1$-methionine) methylated nucleotides from RNA m^{1}A, and m^{5}C in PRPS2 or PRPS1 ectopically expressed H1299 cells. The data in (a-b) were plotted as the mean ± SDs of six biological replicates. *$p < 0.05$ for multiple comparisons, calculated using one-way ANOVA with Tukey's HSD test.

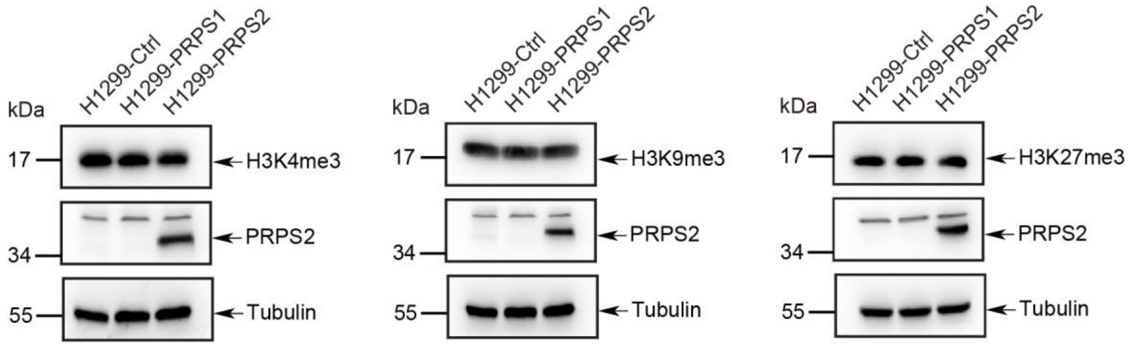

Supplementary Figure 21. Detection of histone H3 trimethylation (H3K4me3, H3K9me3, and H3K27me3) in PRPS2 or PRPS1 ectopically expressed H1299 cells.

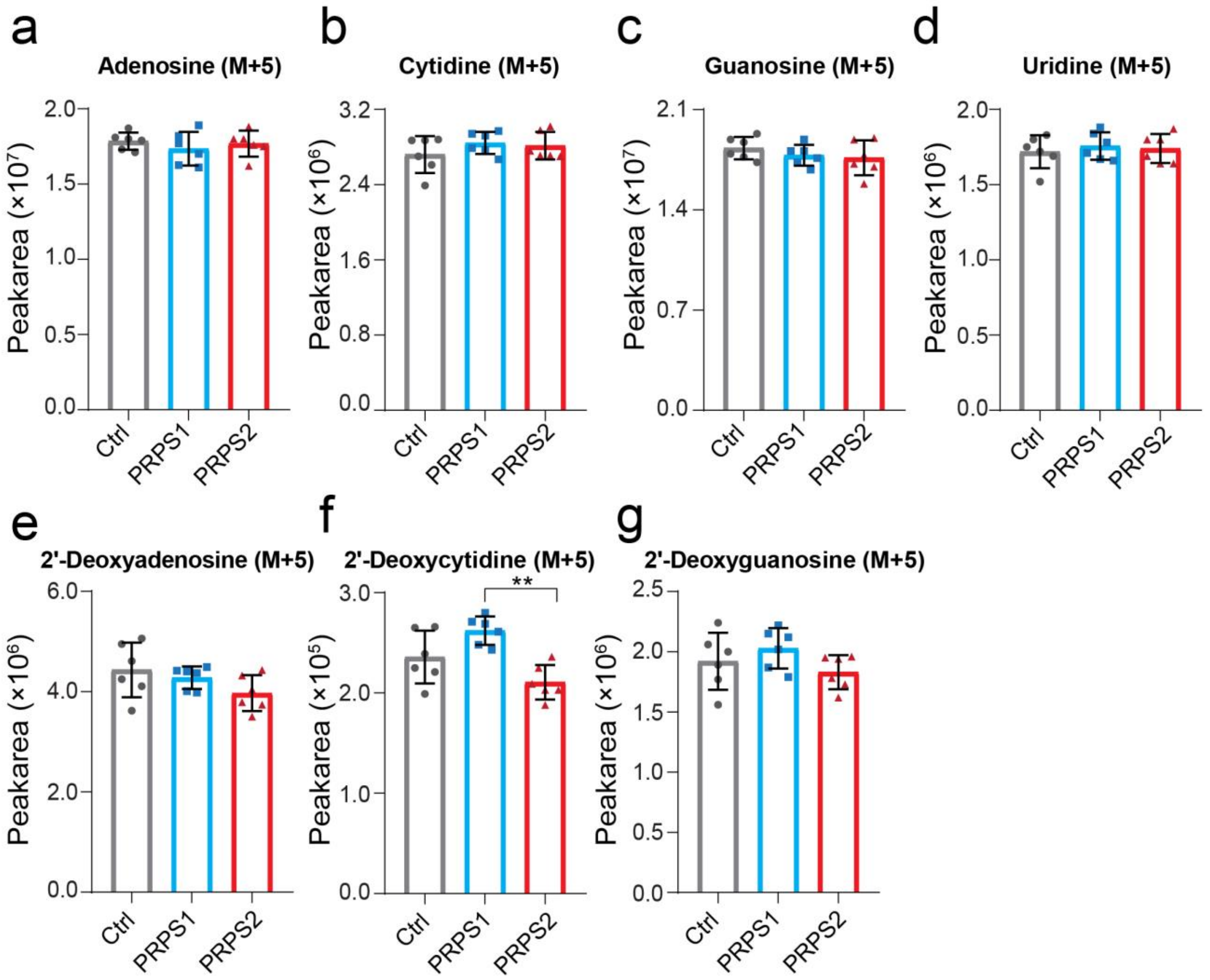

Supplementary Figure 22. Peak areas of ^{13}C-labeled DNA and RNA synthesis measured by targeted LC-MS/MS in PRPS2 ectopically expressed H1299 cells. The data were monitored after a 6-hour timeframe with $^{13}C_6$-glucose (red dots). The data in (a-g) were plotted as the mean ± SDs of six biological replicates. **p < 0.01 for multiple comparisons calculated using one-way ANOVA with Tukey's HSD test.

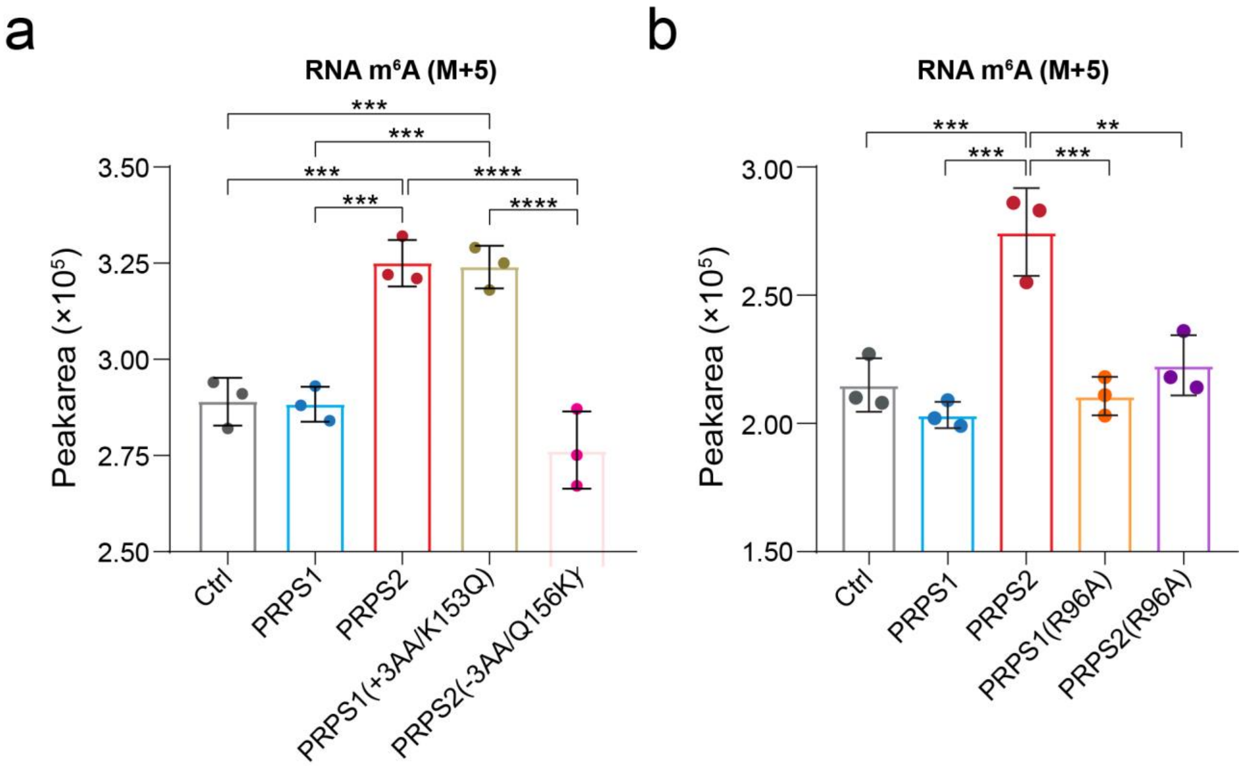

Supplementary Figure 23. Peak areas of ^{13}C-labeled ($^{13}C_6$-glucose) methylated nucleotides from RNA m^{6}A in PRPS1, PRPS2, or their mutant ectopically expressed H1299 cell line. The data were plotted as the mean ± SDs of biological triplicates. **p < 0.01, ***p < 0.001, and ****p < 0.0001 for multiple comparisons calculated using one-way ANOVA with Tukey's HSD test.

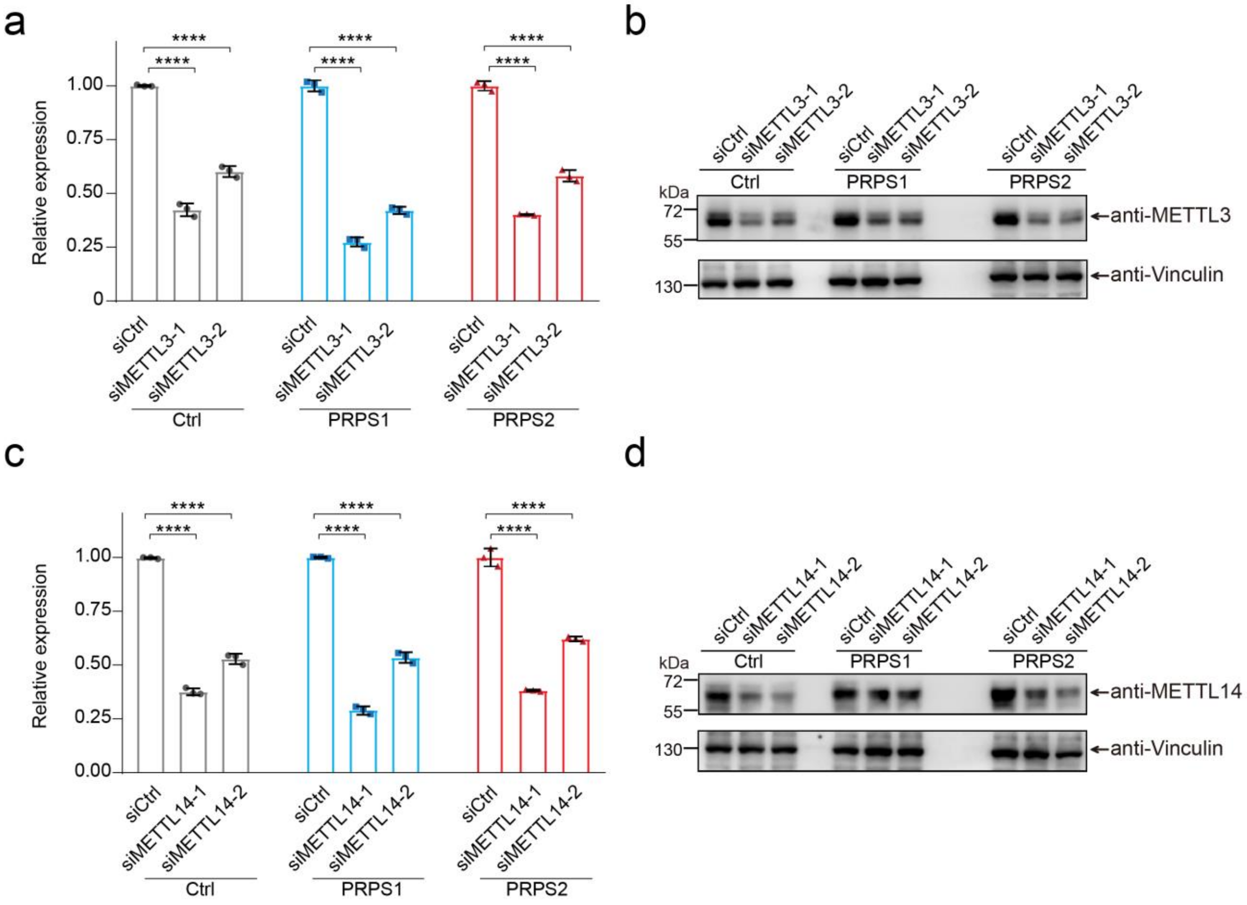

Supplementary Figure 24. Relative expression of METTL3 (a, b) or METTL14 (c, d) in PRPS2 or PRPS1 ectopically expressed H1299 cells treated with siRNA. The data in (a, c) were plotted as the mean ± SDs of biological triplicates. ****$p < 0.0001$ for multiple comparisons calculated using one-way ANOVA with Tukey's HSD test.

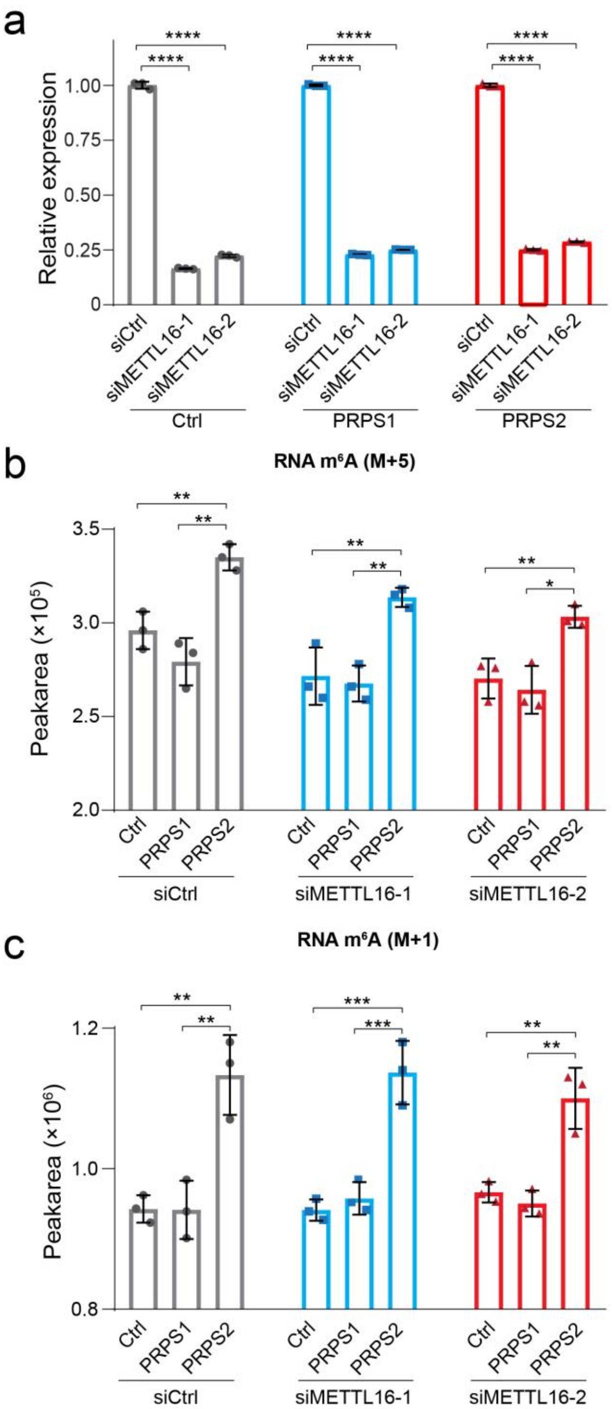

439 **Supplementary Figure 25.** (a) Relative expression of METTL16 in PRPS2 or PRPS1
440 ectopically expressed H1299 cells treated with siRNA. (b-c) Peak areas of ^{13}C-labeled
441 methylated nucleotides from RNA m^{6}A in PRPS2 or PRPS1 ectopically expressed H1299 cells
442 treated with siRNA. The data in (a-b) were plotted as the mean ± SDs of biological triplicates. *p
443 < 0.05, **p < 0.01, ***p < 0.001, and ****p < 0.0001 for multiple comparisons calculated using
444 one-way ANOVA with Tukey's HSD test.

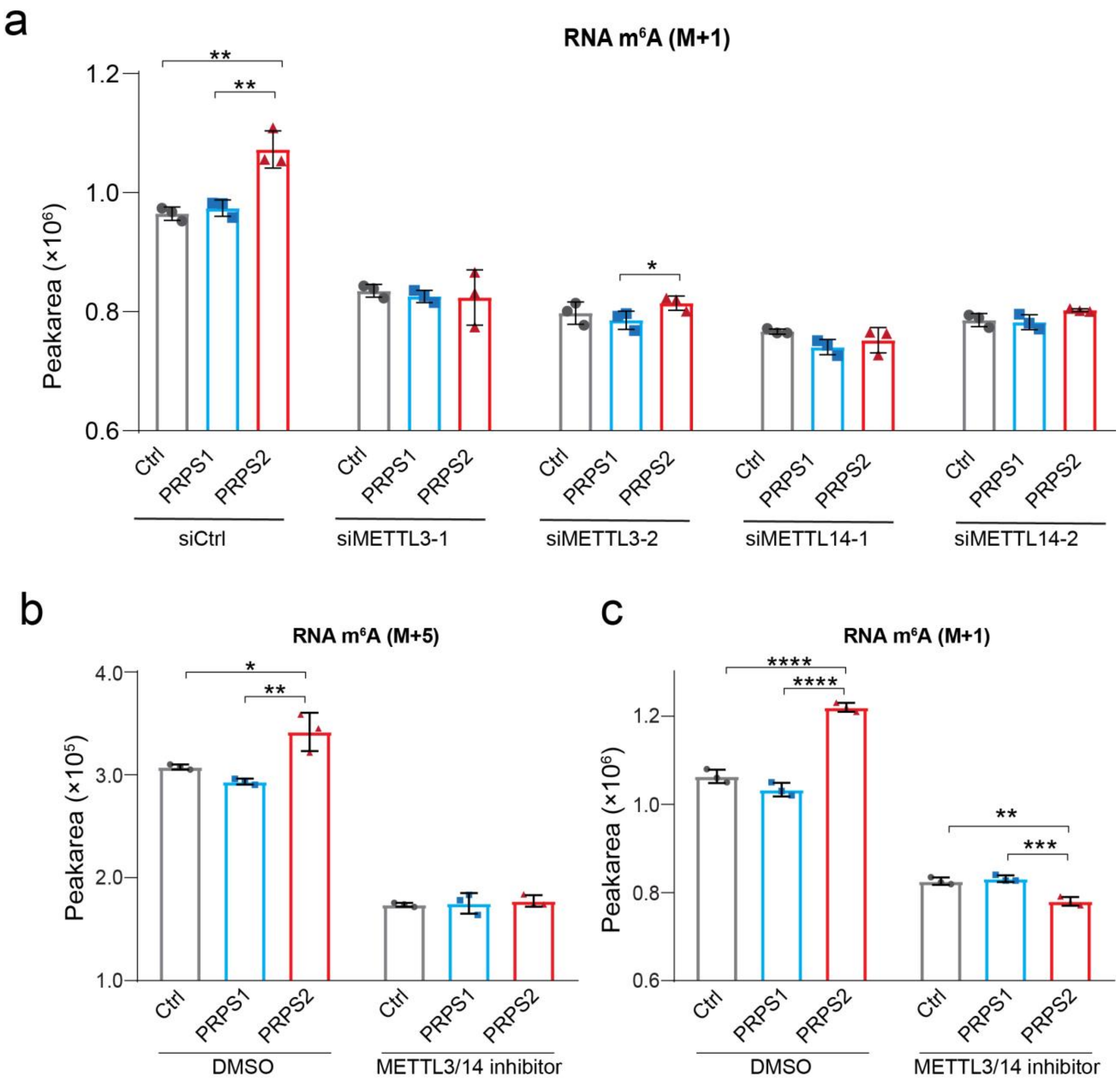

Supplementary Figure 26. (a) Peak areas of ^{13}C-labeled ($^{13}C_1$-methionine) methylated nucleotides from RNA m^{6}A after siRNA knockdown of METTL3 or METTL14 in PRPS2 or PRPS1 ectopically expressed H1299 cell lines. (b-c) Peak areas of ^{13}C-labeled ($^{13}C_6$-glucose or $^{13}C_1$-methionine) methylated nucleotides from RNA m^{6}A upon METTL3/ METTL14 inhibitor STM2457 treatment in PRPS2 or PRPS1 ectopically expressed H1299 cell lines. The data in (a-b) were plotted as the mean ± SDs of biological triplicates. *$p < 0.05$, **$p < 0.01$, ***$p < 0.001$, and ****$p < 0.0001$ for multiple comparisons calculated using one-way ANOVA with Tukey's HSD test.

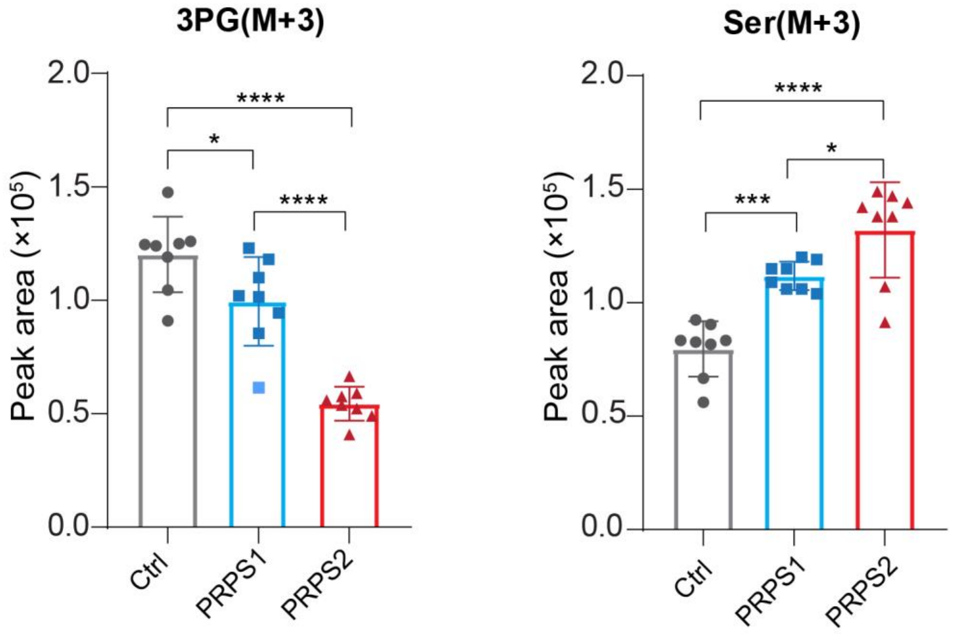

Supplementary Figure 27. Peak areas of ^{13}C-labeled metabolites through the serine biosynthesis pathway measured by targeted LC-MS/MS in PRPS2 ectopic expressed H1299 cells. The data were monitored after a 6-hour timeframe with $^{13}C_6$-glucose. The data in (a-b) were plotted as the mean ± SDs of eight biological replicates. *p < 0.05, ***p < 0.001, and ****p < 0.0001 for multiple comparisons calculated using one-way ANOVA with Tukey's HSD test.
