# Supplementary material for: PRPS2 enhances RNA m6A methylation by stimulating SAM synthesis through enzyme-dependent and independent mechanisms
Source: Nat Commun. 2025 Apr 28;16:3966. doi: 10.1038/s41467-025-59119-0 (PMC12037730; doi:10.1038/s41467-025-59119-0)
Supplement: Supplementary file 2 — Reporting Summary [file 41467_2025_59119_MOESM2_ESM.pdf]

nature portfolio

Corresponding author(s): Hao Wang, Jianxiu Yu, Liang Zhang

Last updated by author(s): Mar 12, 2025

Reporting Summary

Nature Portfolio wishes to improve the reproducibility of the work that we publish. This form provides structure for consistency and transparency in reporting. For further information on Nature Portfolio policies, see our Editorial Policies and the Editorial Policy Checklist.

Statistics

For all statistical analyses, confirm that the following items are present in the figure legend, table legend, main text, or Methods section.

n/a	Confirmed	
☐	☒	The exact sample size (*n*) for each experimental group/condition, given as a discrete number and unit of measurement
☐	☒	A statement on whether measurements were taken from distinct samples or whether the same sample was measured repeatedly
☐	☒	The statistical test(s) used AND whether they are one- or two-sided<br>*Only common tests should be described solely by name; describe more complex techniques in the Methods section.*
☐	☒	A description of all covariates tested
☐	☒	A description of any assumptions or corrections, such as tests of normality and adjustment for multiple comparisons
☐	☒	A full description of the statistical parameters including central tendency (e.g. means) or other basic estimates (e.g. regression coefficient) AND variation (e.g. standard deviation) or associated estimates of uncertainty (e.g. confidence intervals)
☐	☒	For null hypothesis testing, the test statistic (e.g. *F*, *t*, *r*) with confidence intervals, effect sizes, degrees of freedom and *P* value noted<br>*Give P values as exact values whenever suitable.*
☒	☐	For Bayesian analysis, information on the choice of priors and Markov chain Monte Carlo settings
☒	☐	For hierarchical and complex designs, identification of the appropriate level for tests and full reporting of outcomes
☒	☐	Estimates of effect sizes (e.g. Cohen's *d*, Pearson's *r*), indicating how they were calculated

Our web collection on statistics for biologists contains articles on many of the points above.

Software and code

Policy information about availability of computer code

Data collection	The crystal diffraction data were processed with HKL3000v721.3. Quantification of DNA/RNA methylation were acquired by using a UHPLC system (1290 Infinity LC, Agilent Technologies) coupled to Triple Quad 6500 System (AB Sciex 6500).
Data analysis	GraphPad Prism 8; CCP4 7.1; Phenix 1.18; Coot 0.8.9.1; Pymol 1.8.0.3; MicroCal ITC200 Analysis Software; ASTRA 6 software; LightCycler 480 Software; xCELLigence RTCA-DP system software; IncuCyte Live Cell Analysis system; ImageJ;

For manuscripts utilizing custom algorithms or software that are central to the research but not yet described in published literature, software must be made available to editors and reviewers. We strongly encourage code deposition in a community repository (e.g. GitHub). See the Nature Portfolio guidelines for submitting code & software for further information.

Data

Policy information about availability of data

All manuscripts must include a data availability statement. This statement should provide the following information, where applicable:

- Accession codes, unique identifiers, or web links for publicly available datasets
- A description of any restrictions on data availability
- For clinical datasets or third party data, please ensure that the statement adheres to our policy

The atomic coordinates and structure factors for the reported crystal structures have been deposited to the Protein Data Bank (PDB) under the accession codes 8YPY, 8YPZ, and 8YQ0. The online database Gene Expression Profiling Interactive Analysis (GEPIA, http://gepia.cancerpku.cn/index.html) was used to analyze the

RNA sequencing expression of PRPS1 and PRPS2 in lung adenocarcinoma (LUAD) and breast invasive carcinoma (BRCA) patients based on The Cancer Genome Atlas (TCGA) and the Genotype-Tissue Expression (GTEx) projects. The overall survival analyses were performed by using GEPIA web tool, and the recurrence-free survival analyses of the BRCA patients were executed by using KM plotter (http://kmplot.com/analysis/) web tool. The microarray gene expression profiling data sets (GSE33356, GSE43458, GSE10192, GSE7670, GSE32867, GSE1037, GSE75037, GSE27262) were downloaded from Gene Expression Omnibus (GEO, https://www.ncbi.nlm.nih.gov/geo/) database.

Research involving human participants, their data, or biological material

Policy information about studies with human participants or human data. See also policy information about sex, gender (identity/presentation), and sexual orientation and race, ethnicity and racism.

Reporting on sex and gender	Included in Supplementary Table I.
Reporting on race, ethnicity, or other socially relevant groupings	Included in Supplementary Table I.
Population characteristics	Included in Supplementary Table I.
Recruitment	Included in Supplementary Table I.
Ethics oversight	Ethics Committee of Zhongshan Hospital, Fudan University, and performed in accordance with the principles of the Declaration of Helsinki

Note that full information on the approval of the study protocol must also be provided in the manuscript.

Field-specific reporting

Please select the one below that is the best fit for your research. If you are not sure, read the appropriate sections before making your selection.

☒ Life sciences ☐ Behavioural & social sciences ☐ Ecological, evolutionary & environmental sciences

For a reference copy of the document with all sections, see nature.com/documents/nr-reporting-summary-flat.pdf

Life sciences study design

All studies must disclose on these points even when the disclosure is negative.

Sample size	Stated in Methods and/or the respective figure legends.
Data exclusions	No data were excluded from the analysis.
Replication	Stated in Methods and/or the respective figure legends.
Randomization	The endogenous PRPS2 protein levels from 12 pairs of lung adenocarcinoma specimens with adjacent normal tissues were randomly chosen.
Blinding	The histologic types of patients were independently assigned by at least 3 pathologists in a double-blinded fashion. The other samples and experiments were not blinded to the authors, since all measurements were objective and the conclusions were based on multiple independent experiments, technical replicates and statistical significance.

Reporting for specific materials, systems and methods

We require information from authors about some types of materials, experimental systems and methods used in many studies. Here, indicate whether each material, system or method listed is relevant to your study. If you are not sure if a list item applies to your research, read the appropriate section before selecting a response.

Materials & experimental systems

n/a	Involved in the study	
☐	☒	Antibodies
☐	☒	Eukaryotic cell lines
☒	☐	Palaeontology and archaeology
☐	☒	Animals and other organisms
☒	☐	Clinical data
☒	☐	Dual use research of concern
☒	☐	Plants

Methods

n/a	Involved in the study	
☒	☐	ChIP-seq
☒	☐	Flow cytometry
☒	☐	MRI-based neuroimaging

Antibodies

Antibodies used

Antibodies for western blotting:
Alpha Tubulin Monoclonal antibody Protein Tech Group, #66031-1-Ig, 1:1000 ;
Vinculin Monoclonal antibody (Protein Tech Group #66305-1-Ig, 1:1000);
DYKDDDDK Tag Recombinant Rabbit Monoclonal Antibody (invitrogen,701629, 1:1000) ;
HA Tag Monoclonal Antibody(invitrogen,26183,1:2000);
PRPS2 (homemade, 1:1000)
MAT2A Polyclonal antibody (Protein Tech Group, #55309-1-AP, 1:1000)
Beta Actin Monoclonal antibody (Protein Tech Group #66009-1-Ig, 1:1000)
Tri-Methyl-Histone H3 (Lys4) Antibody (CST, #9727,1:1000)
Tri-Methyl-Histone H3 (Lys27) (C36B11) Rabbit mAb (CST, #9733,1:1000)
Tri-Methyl-Histone H3 (Lys9) (D4W1U) Rabbit mAb (CST, #13969,1:1000)
METTL3 (Protein Tech Group, #15073-1-AP, 1:1000)
METTL14 (Protein Tech Group, #26158-1-AP, 1:1000)
Goat anti-Rabbit IgG (H+L) Secondary Antibody, HRP, Thermo Fisher, Cat# 31460, 1:5000;
Goat anti-Mouse IgG (H+L) Secondary Antibody, HRP, Thermo Fisher, Cat# 31430, 1:10000;
Antibodies for immunofluorescence:
Goat anti-Rabbit IgG (H+L) Cross-Adsorbed Secondary Antibody, Alexa Fluor™ 594, invitrogen, A-11012, 1:200;
Donkey anti-Mouse IgG (H+L) Highly Cross-Adsorbed Secondary Antibody, Alexa Fluor™ 647, invitrogen, A-31571, 1:200;

Validation

We developed a PRPS2-specific antibody capable of distinguishing PRPS2 from PRPS1 (Supplementary Figure 2).
Antibodies from commercial sources have been validated by manufacturer with either as proofs or publication references on the manufacturers website.
anti-tubulin:https://www.ptgcn.com/products/tubulin-Alpha-Antibody-66031-1-Ig.htm
anti-vinculin:https://www.ptgcn.com/products/Vinculin-Antibody-66305-1-Ig.htm
anti-flag: https://www.thermofisher.cn/cn/zh/antibody/product/DYKDDDDK-Tag-Antibody-clone-8H8L17-Recombinant-Monoclonal/701629
anti-HA: https://www.thermofisher.cn/cn/zh/antibody/product/HA-Tag-Antibody-clone-2-2-2-14-Monoclonal/26183
anti-MAT2A: https://www.ptgcn.com/products/MAT2A-Antibody-55309-1-AP.htm
anti-actin: https://www.ptgcn.com/products/Pan-Actin-Antibody-66009-1-Ig.htm
anti-H3K4:https://www.cellsignal.cn/products/primary-antibodies/tri-methyl-histone-h3-lys4-antibody/9727
anti-H3K27:https://www.cellsignal.cn/products/primary-antibodies/tri-methyl-histone-h3-lys27-c36b11-rabbit-mab/9733
anti-H3K9:https://www.cellsignal.cn/products/primary-antibodies/tri-methyl-histone-h3-lys9-d4w1u-rabbit-mab/13969
METTL3:https://www.ptgcn.com/products/METTL3-Antibody-15073-1-AP.htm
METTL14:https://www.ptgcn.com/products/METTL14-Antibody-26158-1-AP.htm
Secondary Antibody, Alexa Fluor™ 594:https://www.thermofisher.cn/cn/zh/antibody/product/Goat-anti-Rabbit-IgG-H-L-Cross-Adsorbed-Secondary-Antibody-Polyclonal/A-11012
Secondary Antibody, Alexa Fluor™ 647:https://www.thermofisher.cn/cn/zh/antibody/product/Donkey-anti-Mouse-IgG-H-L-Highly-Cross-Adsorbed-Secondary-Antibody-Polyclonal/A-31571

Eukaryotic cell lines

Policy information about cell lines and Sex and Gender in Research

Cell line source(s)

H1299, Stem Cell Bank, Chinese Academy of Sciences, Cat#TCHu160
A549, Stem Cell Bank, Chinese Academy of Sciences, Cat#TCHu150
H1975,Stem Cell Bank, Chinese Academy of Sciences, Cat#TCHu193
H838,Stem Cell Bank, Chinese Academy of Sciences, Cat#TCHu241
H1792, Stem Cell Bank, Chinese Academy of Sciences, Cat#SCSP-5222
WI38/VA13, Stem Cell Bank, Chinese Academy of Sciences, Cat#GNHu52
H460, Stem Cell Bank, Chinese Academy of Sciences, Cat#TCHu205
H1944,Chinese National Infrastructure of Cell Line Resource (NICR) , Cat#3101HUMSCSP596
H520, Chinese National Infrastructure of Cell Line Resource (NICR) , Cat# 5301HUM-KCB13023YJ
H1650, Chinese National Infrastructure of Cell Line Resource (NICR) , Cat#3101HUMSCSP592
H661, Chinese National Infrastructure of Cell Line Resource (NICR) , Cat#3101HUMTCHu121
PC9 and HCC827 were kindly provided by Dr. Ying Shen from Shanghai Jiao Tong University School of Medicine.

Authentication

None of the cell lines we used were authenticated.

Mycoplasma contamination

No mycoplasma contamination was detected.

Commonly misidentified lines
(See ICLAC register)

None.

Animals and other research organisms

Policy information about studies involving animals; ARRIVE guidelines recommended for reporting animal research, and Sex and Gender in Research

Laboratory animals	6-week-old BALB/c nude mice were maintained in the animal facility of Shanghai Jiao Tong University School of Medicine.
Wild animals	None.
Reporting on sex	All male gender.
Field-collected samples	No field-collected samples were used in this study.
Ethics oversight	The procedures related to animal subjects were approved by the ethics committee of the Department of Laboratory Animal Science, Shanghai Jiaotong University School of Medicine.

Note that full information on the approval of the study protocol must also be provided in the manuscript.

Plants

Seed stocks	*Report on the source of all seed stocks or other plant material used. If applicable, state the seed stock centre and catalogue number. If plant specimens were collected from the field, describe the collection location, date and sampling procedures.*
Novel plant genotypes	*Describe the methods by which all novel plant genotypes were produced. This includes those generated by transgenic approaches, gene editing, chemical/radiation-based mutagenesis and hybridization. For transgenic lines, describe the transformation method, the number of independent lines analyzed and the generation upon which experiments were performed. For gene-edited lines, describe the editor used, the endogenous sequence targeted for editing, the targeting guide RNA sequence (if applicable) and how the editor was applied.*
Authentication	*Describe any authentication procedures for each seed stock used or novel genotype generated. Describe any experiments used to assess the effect of a mutation and, where applicable, how potential secondary effects (e.g. second site T-DNA insertions, mosiacism, off-target gene editing) were examined.*
